# Plumage microorganism communities of tidal marsh sparrows

**DOI:** 10.1016/j.isci.2023.108668

**Published:** 2023-12-07

**Authors:** Alice M. Hotopp, Brian J. Olsen, Suzanne L. Ishaq, Serita D. Frey, Adrienne I. Kovach, Michael T. Kinnison, Franco N. Gigliotti, Mackenzie R. Roeder, Kristina M. Cammen

**Affiliations:** 1 School of Biology and Ecology, University of Maine, Orono, ME 04469, USA; 2 School of Food and Agriculture, University of Maine, Orono, ME 04469, USA; 3 Department of Natural Resources and the Environment, University of New Hampshire, Durham, NH 03824, USA; 4 Department of Ecology and Evolutionary Biology, University of Connecticut, Storrs, CT 06269, USA; 5 School of Marine Sciences, University of Maine, Orono, ME 04469, USA; 6 Maine Center for Genetics in the Environment, University of Maine, Orono, ME 04469, USA

**Keywords:** Ornithology, Microbiology, Evolutionary biology

## Abstract

Microorganism communities can shape host phenotype evolution but are often comprised of thousands of taxa with varied impact on hosts. Identification of taxa influencing host evolution relies on first describing microorganism communities and acquisition routes. Keratinolytic (keratin-degrading) microorganisms are hypothesized to be abundant in saltmarsh sediments and to contribute to plumage evolution in saltmarsh-adapted sparrows. Metabarcoding was used to describe plumage bacterial (16S rRNA) and fungal (ITS) communities in three sparrow species endemic to North America’s Atlantic coast saltmarshes. Results describe limited within-species variability and moderate host species-level patterns in microorganism diversity and community composition. A small percentage of overall microorganism diversity was comprised of potentially keratinolytic microorganisms, warranting further functional studies. Distinctions between plumage and saltmarsh sediment bacteria, but not fungal, communities were detected, suggesting multiple bacterial acquisition routes and/or vertebrate host specialization. This research lays groundwork for future testing of causal links between microorganisms and avian host evolution.

## Highlights

• Plumage microorganism communities vary somewhat across tidal marsh sparrow species
• Within-species variation in microorganism communities is unexplained by ecology
• Keratin-degrading microorganisms inhabit tidal marsh sparrow plumage
• Saltmarsh sediment and plumage bacterial, but not fungal, communities are distinct

## Graphical abstract

**Figure:**
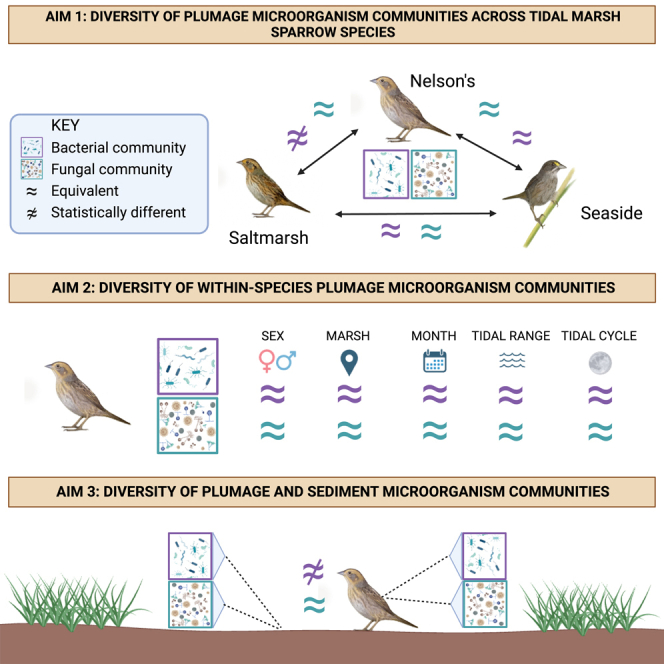

## Introduction

Microbiomes can influence numerous vertebrate functions and phenotypes, including development, digestion, reproduction, immunity, and behavior.^1^ In birds, for example, variation in community composition and/or diversity of microbial communities of avian plumage has been associated with variation in structural coloration,^2^^,^^3^^,^^4^ preen gland size and preening behavior,^5^^,^^6^^,^^7^^,^^8^ feather and body condition,^3^^,^^8^ migratory behavior,^9^ extent of parental investment^10^ and host immunity.^11^ These associations suggest a potential role of microbial communities in shaping avian phenotypes. Yet, it remains difficult to fully understand these animal-microorganism interactions when it is unknown if such associations are the outcome of host-microbe coevolution or simply reflective of patterns of opportunistic colonization.

Microorganism communities of integumentary structures (such as skin, nails, hair, and feathers) are shaped both by host physiology and the external environment;^12^ some members of those communities may in turn influence host phenotypes and fitness.^13^ Microorganism communities can also be comprised of taxa that may vary in their association with hosts and in their functions across space and time.^14^^,^^15^ Therefore, it can be difficult to identify which microorganisms (if any) influence vertebrate host phenotypes and fitness, and how.^16^ Important first steps in the identification of evolutionarily important taxa include comprehensively describing microorganism communities and potential colonization routes, which help to contextualize the nature of association. For example, coevolution of hosts and microorganisms can occur either through vertical transmission of taxa from generation to generation, or through consistent, across-generation acquisition of taxa from the environment.^17^

Here, we describe the feather microbiota of three avian species inhabiting tidal saltmarshes. Our overarching motivation was to provide the foundational data necessary to inform future tests for evolutionarily important taxa and avian-microorganism coevolution. Tidal saltmarshes pose many challenges to inhabitants such as tidal flooding and high salinity, and are therefore home to few endemic vertebrate species.^18^ Amongst these are three species of tidally adapted sparrows within the genus *Ammospiza*. Saltmarshes contain microorganisms that are hypothesized to be selective agents on these saltmarsh-endemic birds,^19^ making these ecosystems good candidates for the study of association between vertebrate hosts and microorganisms. Keratinolytic bacteria, those capable of degrading the beta-keratin proteins that compose avian feathers,^20^ are salt-tolerant and act as plant symbionts that alleviate salt induced stress.^21^^,^^22^ Similarly, many fungal taxa that are decomposers of native saltmarsh plants (e.g., *Aspergillus*, *Fusarium*, *Paecilomyces*, *Penicillium*, *Cadophora*, *Trichoderma*, and *Scopulariopsis*) are also keratinolytic.^23^^,^^24^^,^^25^^,^^26^ These keratinolytic microorganisms may colonize avian plumage as tidal marsh sparrows forage and seek shelter under the vegetation canopy on the floor of the marsh where they are in contact with saltmarsh sediments. In a previous study, swamp sparrows (*Melospiza georgiana*) breeding in tidal marshes were shown to harbor a higher load of keratinolytic bacteria than those in inland, freshwater marshes.^19^ It is unclear, however, whether keratinolytic microorganisms are common members of plumage microbiomes in other tidal marsh sparrows, or whether the plumage of any tidal marsh species possesses a core microbial community with which coevolution might occur.

**Figure 1:**
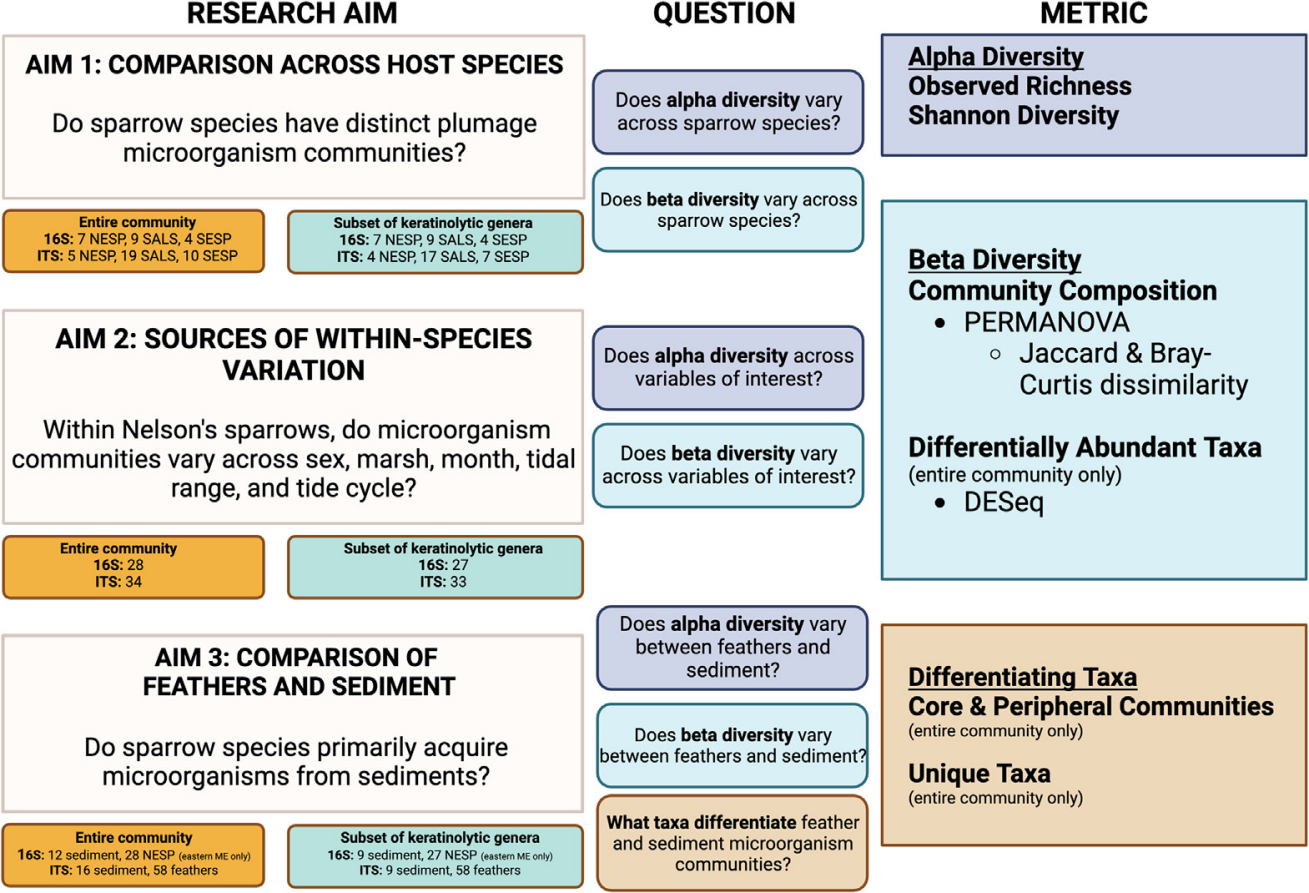
Diagram of this study’s three research aims, sample types and sizes, the specific questions asked within each research aim, and the statistical methods employed

**Figure 2:**
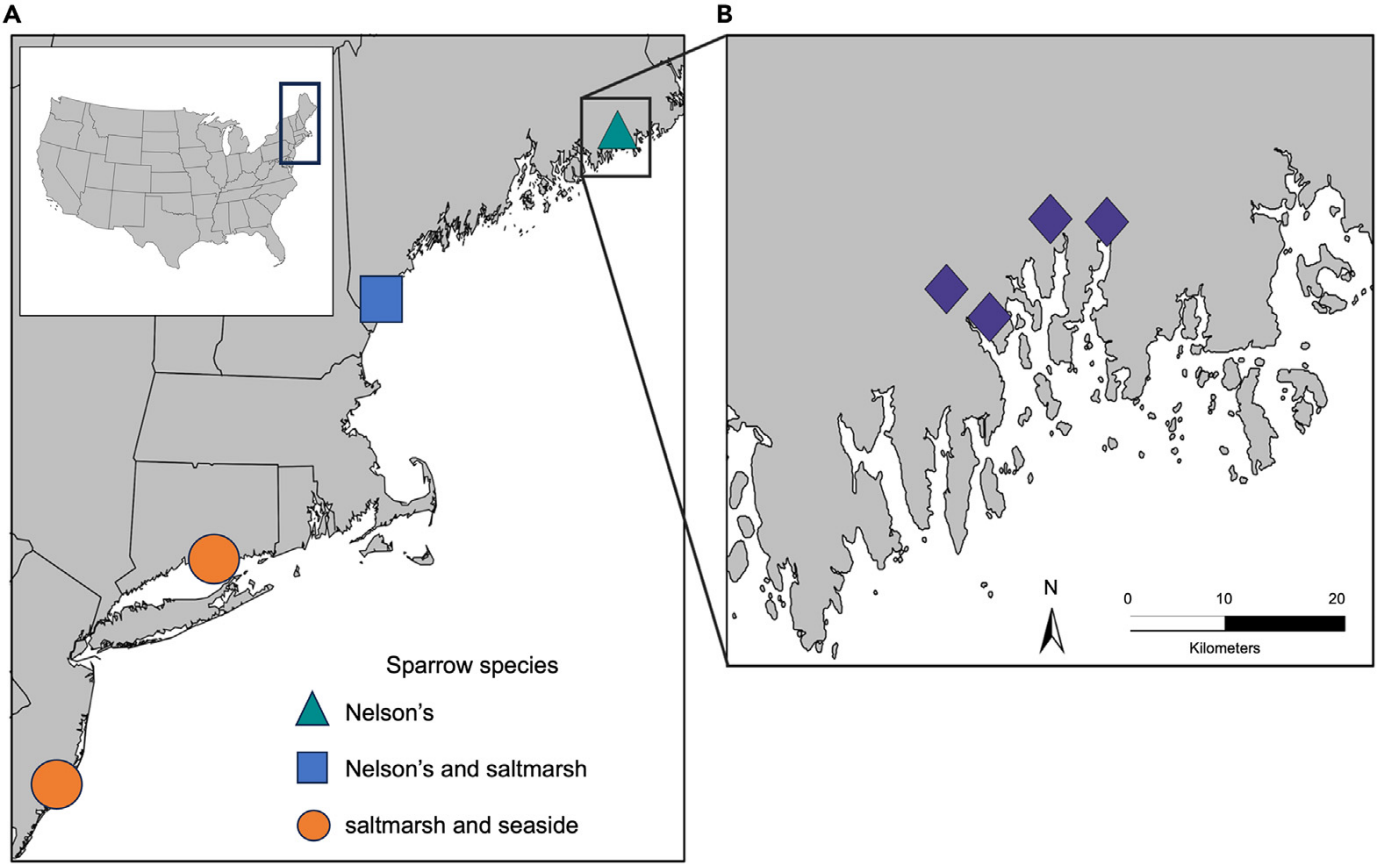
Tidal marsh sparrow plumage sampling sites (A) Sampling sites for Nelson’s, saltmarsh, and seaside sparrow plumage across the Northeast US (Aim 1) in Washington County in eastern Maine; Rachel Carson Wildlife Refuge in Wells, Maine; Hammonasset Beach State Park in Madison, Connecticut; and Edwin B. Forsythe National Wildlife Refuge in Galloway, New Jersey (listed from north to south). Symbols indicate which sparrow species occur at each sampling location. (B) Sampling sites for Nelson’s sparrow plumage across marshes along the Pleasant River, Harrington River, Beaver Meadow Brook, and the Narraguagus River in Washington County in eastern Maine (listed from east to west; Aim 2). Sediment samples were collected from across all sampling sites (Aim 3). See STAR Methods for a description of the sampling conducted for each research aim.

In this study, we used bacterial and fungal metabarcoding to characterize plumage microorganisms of the Acadian subspecies of the Nelson’s sparrow (*Ammospiza nelsoni subvirgata*), the saltmarsh sparrow (*Ammospiza caudacuta*), and the seaside sparrow (*Ammospiza maritima*). The aims of our study were to (1) determine if and how microorganism diversity and community composition varies across the three sparrow host species; (2) determine how host and environmental factors drive community variation within one species (the Nelson’s sparrow) and one geographic area (eastern Maine); and (3) compare plumage microorganism communities to *in situ* saltmarsh sediment communities, to investigate a potential route of microbial colonization of these ground-foraging and ground-nesting avian taxa (aims summarized in Figure 1; sampling locations for all aims in Figure 2). We described each of these microbial communities broadly and with particular attention paid to genera with reported keratinolytic taxa.

## Results

### Summary statistics

#### Interspecific: Plumage samples from across host species

Feather samples from 20 individuals (saltmarsh sparrows, n *= 9*; Nelson’s sparrows, n *= 7*; seaside sparrows, n *= 4;* Table 1) sampled during the June spring tide had sufficient 16S rRNA sequence data (>1,000 reads) for analysis. The unrarefied interspecific bacterial dataset was comprised of 137,495 sequences, an average of 6,875 sequences per sample, and 1,714 SVs. The rarefied dataset contained 30,300 sequences, an average of 1,515 sequences per sample, and 1,311 SVs. The subset of potentially keratinolytic bacterial taxa contained 2,464 sequences, an average of 123 sequences per sample, and 59 SVs.

**Table 1:** Final sample sizes of bacterial and fungal samples retained for analyses of plumage microorganism community variation across sparrow host species (Aim 1), within eastern Maine Nelson’s sparrow plumage samples (Aim 2), and between plumage and sediment microorganism communities (Aim 3)

	Eastern ME: Pleasant River	Eastern ME: Harrington River	Eastern ME: Narraguagus River	Eastern ME: Beaver Meadow River	Rachel Carson NWR, ME	Hammonasset Beach State Park, CT	Edwin B. Forsythe NWR, NJ
**Aim 1**^a^							

Nelson’s sparrow							
*Bacteria*	0	1	6	0	0	0	0
*Fungi*	0	0	3	0	2	0	0
Saltmarsh sparrow							
*Bacteria*	0	0	0	0	3	1	5
*Fungi*	0	0	0	0	4	8	7
Seaside sparrow							
*Bacteria*	0	0	0	0	0	0	4
*Fungi*	0	0	0	0	0	2	8

**Aim 2**							

Nelson’s sparrow							
*Bacteria*	1	7	18	2	NA	NA	NA
*Fungi*	4	9	19	2	NA	NA	NA

**Aim 3**^a^							

Sediment							
*Bacteria*	1	5	3	3	0	0	0
*Fungi*	1	3	3	1	4	1	2

a For Aims 1 and 3, samples from the eastern Maine marshes were combined in analyses to represent the region as a whole.

The interspecific ITS feather dataset included 34 samples from 34 individuals (saltmarsh sparrows, n *=* 19; Nelson’s sparrows, n *=* 5; seaside sparrows, n *=* 10; Table 1). Prior to rarefying, the dataset contained 222,967 sequences, an average of 6,558 sequences per sample, and 2,934 SVs; the rarefied dataset contained 36,176 sequences, an average of 1,064 sequences per sample, and 2,314 SVs. The subset of potentially keratinolytic fungal taxa contained 1,218 sequences, an average of 43 sequences per sample, and 62 SVs.

#### Intraspecific: Eastern Maine Nelson’s sparrow plumage samples

Following data filtering, quality control, and merging of resequenced samples, 28 samples from 24 Nelson’s sparrow individuals sampled in eastern Maine had sufficient 16S rRNA sequence data (>1,000 reads) for analysis (Table 1). Four of these individuals (17%) were first captured during the June spring tide and then recaptured later in the summer. Samples from recaptured birds were included as independent samples in analyses following the logic and initial findings outlined in the methods. Prior to rarefying, the intraspecific bacterial dataset contained 217,584 sequences, an average of 7,771 sequences per sample, and 2,559 SVs. The rarefied dataset was comprised of 37,100 sequences, an average of 1,325 sequences per sample, and 1,928 SVs. The subset of potentially keratinolytic bacterial taxa contained 2,222 sequences, an average of 82 sequences per sample, and 76 SVs.

Similarly, 34 feather samples from 30 Maine Nelson’s sparrow individuals were included in ITS analyses (Table 1), including three recaptured individuals (one bird was recaptured twice, and is represented in three different sampling events). The unrarefied intraspecific fungal dataset contained 562,434 sequences, an average of 16,542 sequences per sample, and 5,425 SVs. The rarefied dataset contained 36,176 sequences, 1,064 sequences per sample, and 3,597 SVs. The subset of potentially keratinolytic fungal taxa contained 1,693 sequences, an average of 51 sequences per sample, and 109 SVs.

#### Eastern Maine sediment samples

A total of 12 sediment samples collected in Maine were retained for 16S rRNA analyses (Table 1), containing 420,826 sequences, an average of 35,069 reads per sample, and 6,266 SVs in the unrarefied dataset. When rarefied to the minimum read depth, the sediment dataset contained 71,676 sequences, an average of 5,973 sequences per sample, and 5,532 SVs. The subset of potentially keratinolytic bacterial taxa contained 408 sequences, an average of 41 sequences per sample, and 29 SVs.

Only 16 sediment samples met the criteria of containing >1,000 ITS sequences to be retained for analysis (Table 1). These sediment samples consisted of 198,586 sequences, an average of 12,4112 sequences per sample, and 1,843 SVs prior to rarefying. The rarefied dataset contained 22,512 sequences, 1,407 sequences per sample, and 1,207 SVs. The subset of potentially keratinolytic fungal taxa contained 211 sequences, an average of 23 sequences per sample, and 22 SVs.

### Aim 1: Variation in alpha diversity and community composition across sparrow host species

**Figure 3:**
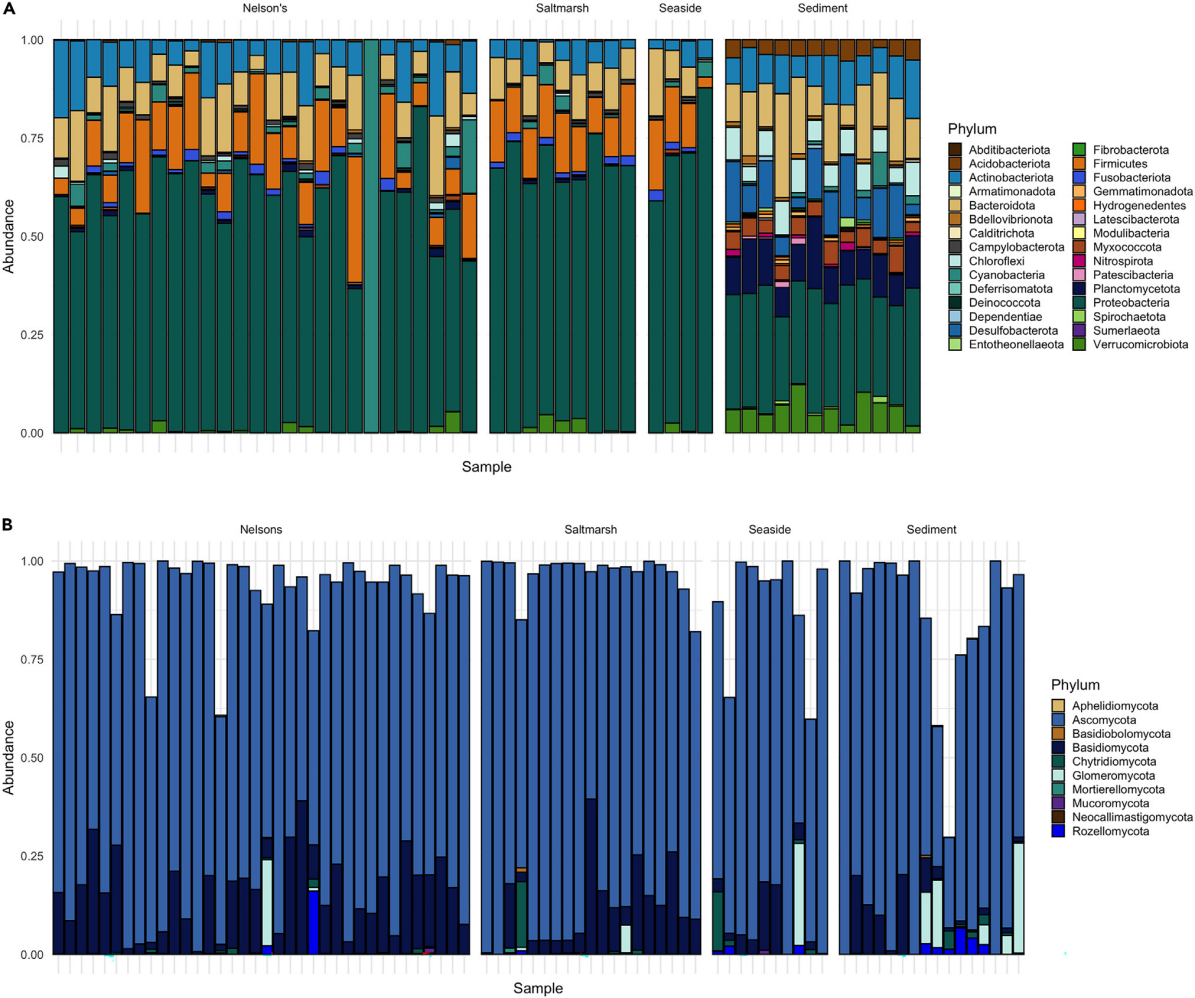
Relative bacterial and fungal taxonomic abundance across feather and sediment samples Relative abundance of (A) bacterial phyla and (B) fungal phyla found in the plumage microorganism communities across all feather samples from *Ammospiza* sparrow host species and concurrently collected sediment samples (samples include those collected for both inter- and intraspecific comparisons). Empty sections of bars in the fungal taxonomy plot represent the percentage of SVs in a sample that were unassigned to a Phylum.

Feather samples collected across host species on the June spring tide contained 19 bacterial phyla, the most abundant of which were Proteobacteria (37.8%), Bacteroidota (22.2%), Actinobacteriota (10.5%), Firmicutes (6.2%), and Planctomycetota (5%). Cumulatively, these top five phyla account for 81.6% of all taxa in the spring tide samples (Figure 3A).

The majority of identified fungal SVs in the June spring tide feather samples belonged to Ascomycota (64.4%) and Basidiomycota (21.6%). A small percentage (2.9%) of SVs were members of Chytridiomycota, Glomeromycota, Rozellomycota, Mucoromycota, Basidiobolomycota, and Mortierellomycota; 11.1% could not be assigned below the level of Kingdom Fungi (Figure 3B).

**Figure 4:**
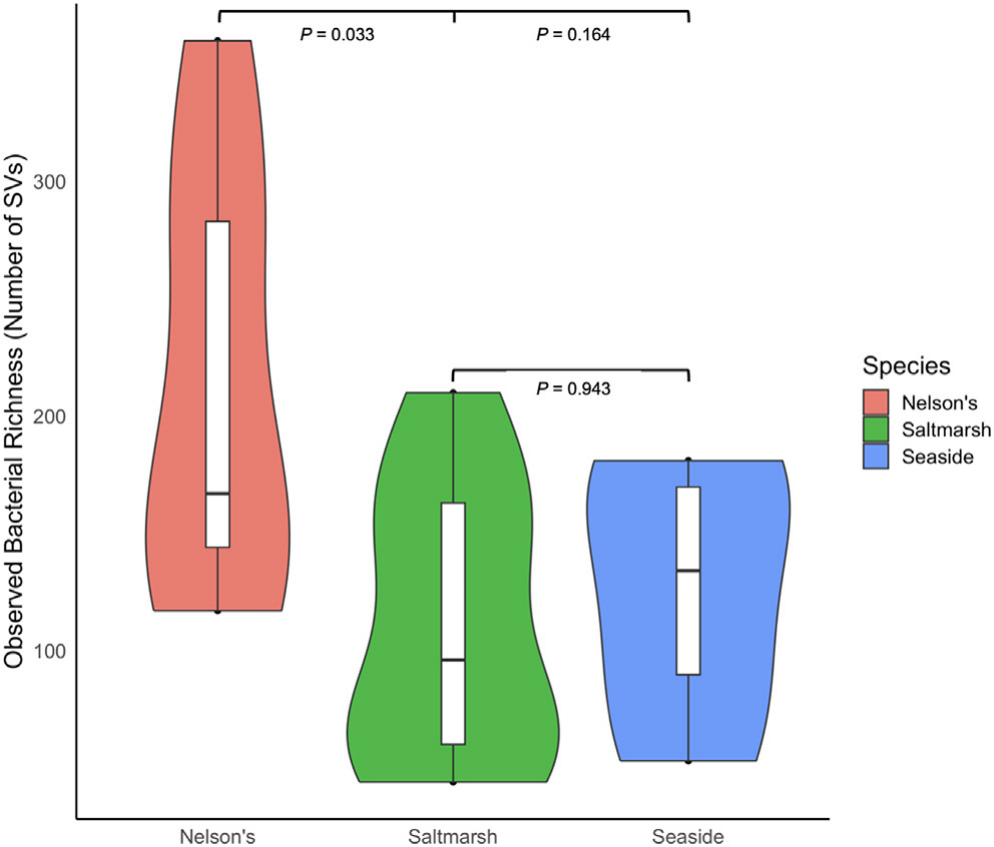
Observed bacterial diversity across tidal marsh sparrow host species Violin plot of interspecific observed richness of plumage bacterial SVs across Nelson’s (n *=* 7), saltmarsh (n *=* 9), and seaside (n *=* 4) sparrows. This metric is significantly higher in Nelson’s sparrows than in saltmarsh sparrows (TukeyHSD, p *=* 0.033). Data are presented as the median value, 1^st^ and 3^rd^ quartiles, and maximum and minimum values.

Variation in alpha diversity across host species was tested using Analysis of Variance (ANOVA) for normally distributed data and using Kruskal-Wallis tests for non-normally distributed data. Significant variation in overall observed richness of bacterial taxa was detected across host species (ANOVA, *F*_2,17_ *=* 4.15, p *=* 0.034). Pairwise Tukey’s honestly significant difference tests (with p values adjusted for multiple comparisons) demonstrated that Nelson’s sparrows had higher richness than saltmarsh sparrows (TukeyHSD, p *=* 0.033; Figure 4).

**Figure 5:**
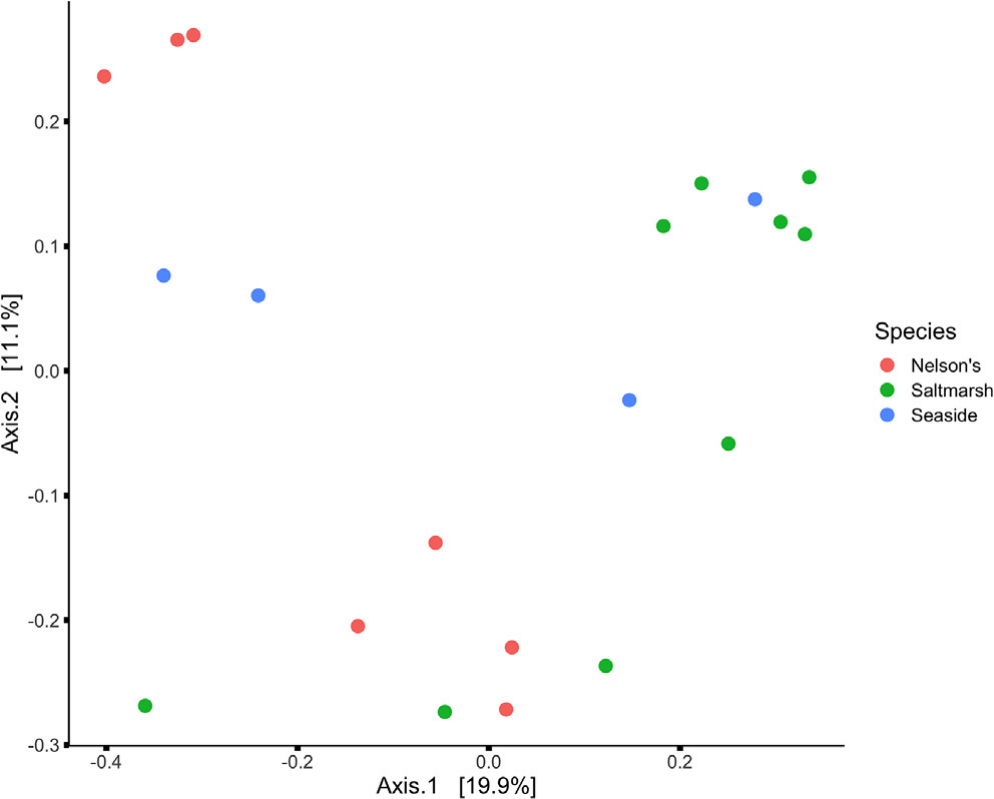
Bacterial community composition across tidal marsh sparrow host species PCoA using Jaccard dissimilarity of plumage bacterial communities across *Ammospiza* sparrow host species (Nelson’s: n *=* 7; saltmarsh: n *=* 9; seaside: n *=* 4). Bacterial community composition significantly differs between Nelson’s and saltmarsh sparrows when calculated using Jaccard dissimilarity (pairwise PERMANOVA, *R*^*2*^*=* 0.129*, F =* 1.4, p *=* 0.035).

Differences in group dispersion and community composition across host species were assessed using Permutational tests (PERMUTEST) and Permutational Analysis of Variance (PERMANOVA), respectively. Host species identity did not affect variance in group dispersion (PERMUTEST, Jaccard: *F*_2,17_ *=* 0.424, p *=* 0.649; Bray-Curtis: *F*_2,17_ *=* 1.55, p *=* 0.067, respectively). However, it did have a significant effect on bacterial community composition when evaluated as presence/absence of taxa (Figure 5; PERMANOVA, Jaccard: *R*^*2*^ *=* 0.141*, F =* 2.06, p *=* 0.03), but not relative abundance (PERMANOVA, Bray-Curtis: *R*^*2*^ *=* 0.154*, F =* 2.17, p *=* 0.613). Nelson’s and saltmarsh sparrows were found to have significantly different community composition using Jaccard dissimilarity (pairwise PERMANOVA, *R*^*2*^ *=* 0.129*, F =* 1.4, p *=* 0.035). A small number of differentially abundant SVs, all within Class Gammaproteobacteria, were detected by DESeq^27^ between each pair of host species (Table 2).

**Table 2:** Bacterial and fungal taxa detected by DESeq at differential abundances in pairwise comparisons of tidal marsh sparrow host species

Host species comparison	SVs more abundant in host species 1	SVs more abundant in host species 2	Absolute value log2fold change	BH corrected p value
*Bacteria: Nelson’s* versus *seaside*	*Marinobacter* SV	*Rickettsiella* SV	24.86 24.78	7.2E-47 6.2E-11
*Bacteria: Nelson’s* versus *saltmarsh*	*Marinobacter* SV *Pseudoalteromonas* SV	*Rickettsiella* SV	24.86 20.78 24.78	7.2E-47 2.2E-07 6.2E-11
*Bacteria: Saltmarsh* versus *seaside*		*Rickettsiella* SV	25.95	6.1E-08
*Fungi: Nelson’s* versus *seaside*	*Paraphaeosphaeria angularis* Ascomycota SV Pleosporales SV Teratosphaeroaceae SV *Phaeosphaeria halima* Neodevriesiaceae SV		21.93 21.39 21.28 21.10 20.53 19.90	2.2E-09 5.7E-05 1.1E-06 2.8E-06 2.2E-05 3.6E-06
*Fungi: Nelson’s* versus *saltmarsh*	*Paraphaeosphaeria angularis* Ascomycota SV Pleosporales SV Teratosphaeriaceae SV *Phaeosphaeria halima* *Alternaria* SV *Dinemasporium* SV Neodevriesiaceae SV Neodevriesiaceae SV *Penicillium* SV Lophiostomataceae SV		21.93 21.39 21.28 21.10 20.53 20.17 20.10 19.90 19.34 19.02 16.78	2.2E-09 5.7E-05 1.0E-06 2.8E-06 2.1E-05 1.9E-04 1.9E-04 3.6E-06 3.9E-04 5.0E-04 4.4E-03
*Fungi: Saltmarsh* versus *seaside*		Ascomycota SV *Phaeosphaeria halima* *Alternaria* SV *Dinemasporium* SV Neodevriesiaceae SV *Penicillium* SV *Parasarocladium* SV	33.78 32.48 30.62 26.95 24.59 23.64 21.30	6.1E-21 8.4E-23 3.9E-17 4.1E-13 8.1E-11 4.7E-10 2.3E-10

Significance was assessed at a Benjamin-Hochberg adjusted p value of <0.01.

Within the subset of genera containing keratinolytic bacterial taxa, no variation in alpha diversity (Observed richness: ANOVA, *F*_2,17_ *=* 1.78, p *=* 0.2; Shannon’s diversity: ANOVA, *F*_2,17_ *=* 1.88, p *=* 0.18). No variation in group dispersion (PERMUTEST, Jaccard: *F*_*2,17*_ *=* 0.255, p *=* 0.77; Bray-Curtis: *F*_*2,17*_ *=* 0.286, p *=* 0.729) or community composition was detected across host species (PERMANOVA, Jaccard: *R*^*2*^ *=* 0.15*, F =* 1.5, p *=* 0.059; Bray-Curtis: *R*^*2*^ *=* 0.15*, F =* 1.5, p *=* 0.12).

Fungal communities did not vary in either observed richness or Shannon diversity across *Ammospiza* host species (ANOVA, *F*_*2,31*_ *=* 1.5*;* p *=* 0.248; Kruskal-Wallis, *X*^*2*^ = 3.977, p *=* 0.137, respectively). When assessed using Jaccard dissimilarity, sparrow host species varied significantly in group dispersion (PERMUTEST, *F*_*2,31*_ = 10.87, p *=* 0.003), likely related to the small Nelson’s sparrow sample size (n *=* 7). However, when assessed using Bray-Curtis dissimilarity, host species fungal community group dispersion did not vary significantly (PERMUTEST, *F*_*2,31*_ *=* 2.3, p *=* 0.12). Fungal community composition appeared to vary significantly among host species (PERMANOVA, *R*^*2*^ *=* 0.07, *F*_*2,31*_ = 1.2, p *=* 0.014), though no species pairs had significantly different community composition after correcting for multiple comparisons. Several SVs were found to be differentially abundant between pairs of host species (Table 2).

Within the subset of keratinolytic fungal genera, alpha diversity did not vary across host species (Observed diversity: Kruskal-Wallis, *X*^*2*^ = 5.89, p *=* 0.05; Shannon’s diversity: ANOVA, *F*_*2,25*_ *=* 2.96*;* p *=* 0.07). Homogeneity of variance of differed significantly between host species (PERMUTEST, Jaccard: *F*_*2,25*_ = 17.24, p *=* 0.001; Bray-Curtis: *F*_*2,25*_ = 5.14, p *=* 0.014), likely due to the small sample size of Nelson’s sparrow feather samples.

### Aim 2: Factors influencing intraspecific variation in alpha diversity and community composition

The bacterial communities detected on the eastern Maine Nelson’s sparrow feathers consisted of 24 phyla and were dominated by Proteobacteria (34.3%), Bacteroidota (23.9%), Actinobacteria (9.8%), Planctomycetota (5.7%) and Verrucomicrobiota (4.7%). These phyla comprised 79.6% of all taxa present in Maine Nelson’s sparrow feathers (Figure 3A). Individual samples displayed a high degree of dissimilarity from one another as demonstrated by the wide scatter of feather samples in a PCoA (Figure S2). Across all bacterial taxa and across the subset of genera containing keratinolytic taxa, no significant variation in observed richness or Shannon diversity was detected across sexes, marshes, month in the breeding season, tidal cycle, or tidal range (Tables S1–S5).

The majority of fungal SVs identified in the eastern Maine Nelson’s sparrow feather samples belonged to the phyla Ascomycota (61.9%) and Basidiomycota (25.5%) (Figure 3B). Taxa belonging to Rozellomycota, Chytridiomycota, Glomeromycota, Mucoromycota, Basidiobolomycota, Mortierellomycota, and Aphelidiomycota collectively comprised 1.7% of SVs; 10.8% were unassigned to taxonomy below the level of Kingdom Fungi. No statistically significant variation in alpha diversity was detected across sexes, marshes, month in the breeding season, tidal range, and tidal cycle in either the entire fungal community or in the subset of genera containing keratinolytic SVs (Tables S6–S9).

Bacterial community group dispersion and community composition did not differ significantly by sex, month, or tidal range under Jaccard dissimilarity (Table S10). However, group dispersion differed significantly across marshes and tidal cycle for both Jaccard and Bray-Curtis dissimilarity. Differentially abundant bacterial SVs were detected between pairwise comparisons of the levels within marsh, tidal cycle, and tidal range (Table S11).

In the subset of bacterial genera containing keratinolytic taxa, homogeneity of variance differed significantly across month, marsh, and tidal range for both metrics of dissimilarity. Across these variables, the groups with the smallest sample sizes tended to have significantly lower dispersions. Bacterial community composition did not vary significantly between sexes (PERMANOVA, Jaccard: *R*^*2*^ = 0.041, *F*_*1,25*_ = 1.07, p *=* 0.355; Bray-Curtis: *R*^*2*^ = 0.044, *F*_*1,25*_ = 1.14, p *=* 0.293) or across tidal cycles (PERMANOVA Jaccard: *R*^*2*^ = 0.077, *F*_*2,24*_ = 0.99, p *=* 0.463; Bray-Curtis: *R*^*2*^ = 0.072, *F*_*2,24*_ = 0.926, p *=* 0.553).

Fungal community composition varied significantly in homogeneity of variance across sex, month, marsh, and tidal cycle for both metrics of dissimilarity. Tidal range differed in homogeneity of variance when assessed with Jaccard, but not Bray-Curtis dissimilarity. Community composition (performed with Bray-Curtis dissimilarity) did not differ significantly across tidal range (PERMANOVA, *R*^*2*^ = 0.095, *F*_*3,30*_ *=* 1.05, p *=* 0.164). Several differentially abundant bacterial SVs were detected between pairwise comparisons of levels within sex, marsh, month, tidal cycle, and tidal range (Table S11).

In the subset of fungal genera containing keratinolytic taxa, homogeneity of variance differed significantly between sexes (PERMUTEST, Jaccard: *F*_*1,31*_ *=* 5.15, p *=* 0.039; Bray-Curtis: *F*_*1,31*_ *=* 4.02, p *=* 0.039). No differences in homogeneity of variance or community composition were detected across month, marsh, tidal cycle, or tidal range with either Jaccard or Bray-Curtis dissimilarity (Table S12).

### Aim 3: Comparison of plumage and sediment microorganism communities

#### Alpha diversity and community composition

Eastern Maine sediment samples contained taxa belonging to 32 bacterial phyla. The most abundant phyla were Proteobacteria (22.8%), Bacteroidota (16.3%), Planctomycetota (12.8%), Verrucomicrobiota (11.2%), and Myxococcota (6.2%). These dominant Phyla accounted for 69.3% of all sequence variants (Figure 3A).

Just over half of the fungal sequence variants present in sediment samples belonged to the phylum Ascomycota (52.9%) (Figure 3B). Variants belonging to Basidiomycota comprised 12% of taxa, and 8.7% of variants were members of Rozellomycota, Glomeromycota, Chytridiomycota, Neocallimastigomycota, Mucoromycota, Basidiobolomycota, and Aphelidiomycota. Unassigned SVs comprised 26.3% of the fungal community.

**Figure 6:**
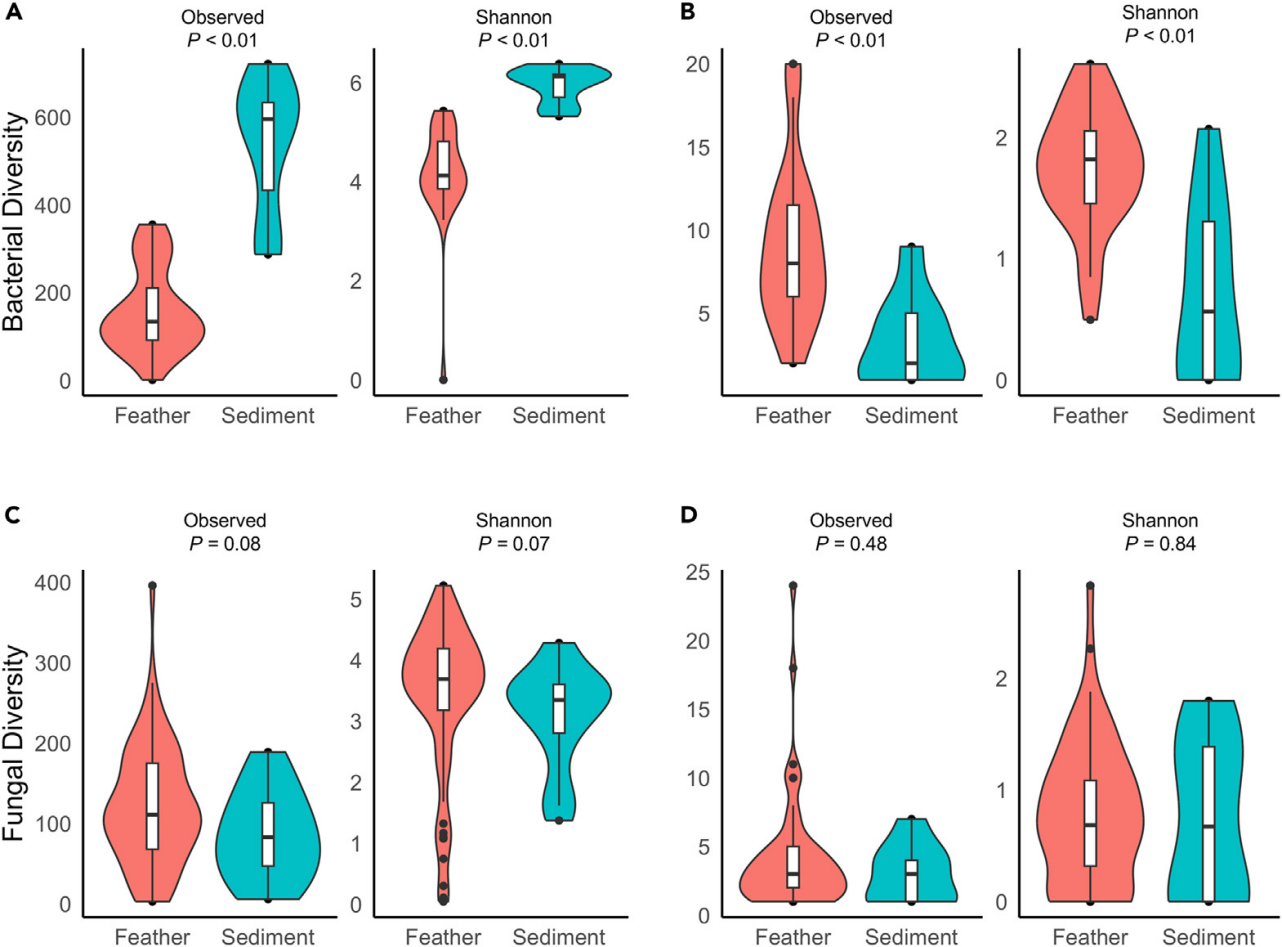
Bacterial and fungal alpha diversity between feather and sediment samples Observed richness and Shannon diversity of feather and sediment samples for (A) entire bacterial communities, (B) a subset of bacterial genera that contain keratinolytic taxa, and (C) across entire fungal communities, and (D) across a subset of fungal genera that contain keratinolytic taxa. Overall bacterial communities varied significantly in both observed richness (Kruskal-Wallis, *X*^*2*^ = 21.12, p *<* 0.01) and Shannon’s diversity (Kruskal-Wallis, *X*^*2*^ = 24, p *<* 0.01). The subsect of genera with keratinolytic taxa also varied significantly in observed richness (Kruskal-Wallis, *X*^*2*^ = 14.81, p *<* 0.01) and Shannon’s diversity (Kruskal-Wallis, *X*^*2*^ = 14.84, p *<* 0.01). Data are presented as the median value, 1^st^ and 3^rd^ quartiles, and maximum and minimum values.

Across all bacterial taxa, eastern Maine sediment samples had significantly higher observed richness (Figure 6A; Kruskal-Wallis, *X*^*2*^ = 21.12, p *<* 0.01) and Shannon’s diversity (Kruskal-Wallis, *X*^*2*^ = 24, p *<* 0.01) than did eastern Maine Nelson’s sparrow feather samples. However, observed richness (Kruskal-Wallis, *X*^*2*^ = 14.81, p *<* 0.01) and Shannon’s diversity (Kruskal-Wallis, *X*^*2*^ = 14.84, p *<* 0.01) of potentially keratinolytic taxa were higher in feather samples than in sediment (Figure 6B).

For fungal communities, feather and sediment samples did not differ significantly in either observed richness or Shannon’s diversity (Figure 6C; Kruskal-Wallis, *X*^*2*^ *=* 3.04, p *=* 0.08; Kruskal-Wallis, *X*^*2*^ *=* 3.34, p *=* 0.07, respectively). Observed richness and Shannon’s diversity also did not vary across sample types within the subset of potentially keratinolytic fungal taxa (Figure 6D; Kruskal-Wallis, *X*^*2*^ *=* 0.49, p *=* 0.48; Kruskal-Wallis, *X*^*2*^ *=* 0.04, p *=* 0.84, respectively).

**Figure 7:**
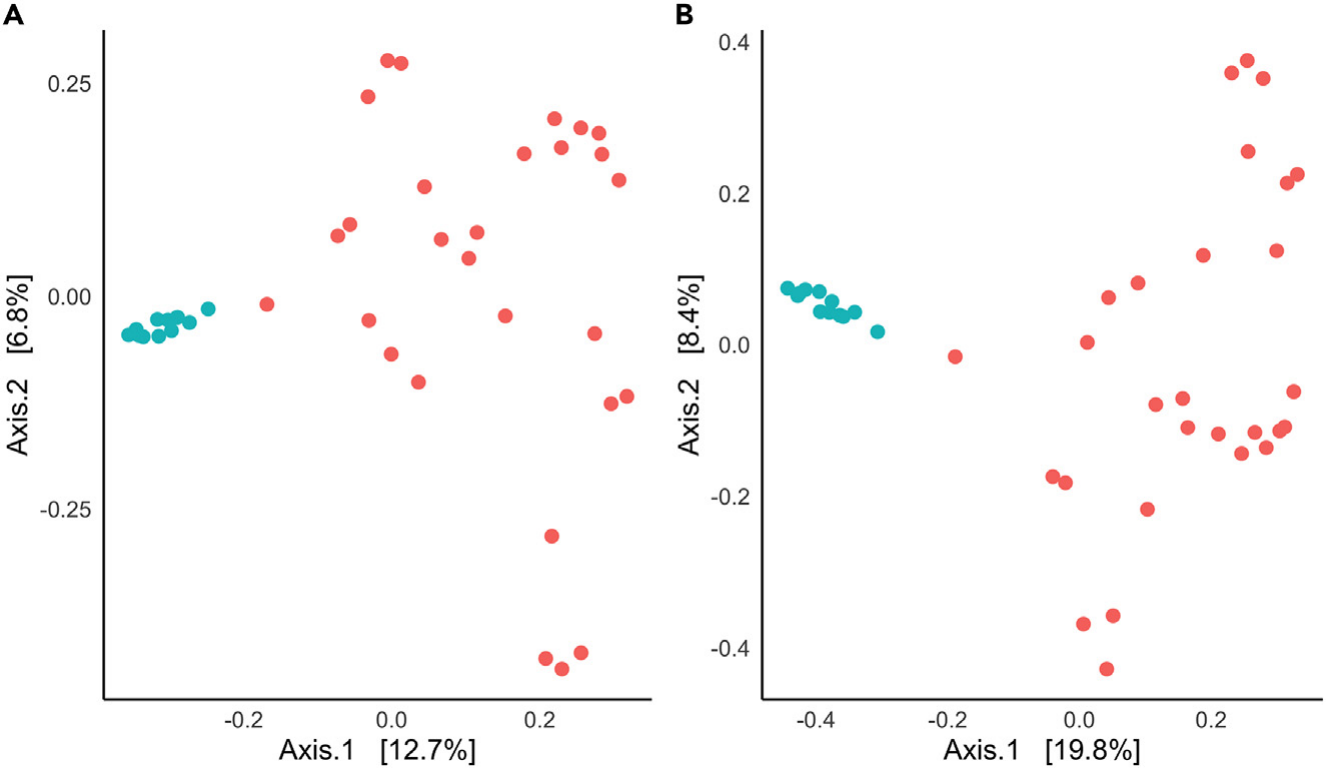
Bacterial community composition of feather and sediment samples PCoA demonstrating distinct bacterial community composition between eastern Maine Nelson’s sparrow feather samples (red, n *=* 28) and eastern Maine sediment samples (blue, n *=* 12) when calculated with (A) Jaccard (PERMANOVA, *R*^*2*^*=* 0.12, *F*_*1,38*_*=* 4.56, p *=* 0.001) and (B) Bray-Curtis (PERMANOVA, *R*^*2*^*=* 0.174, *F*_*1,38*_*=* 8.016, p *=* 0.001) dissimilarity.

**Figure 8:**
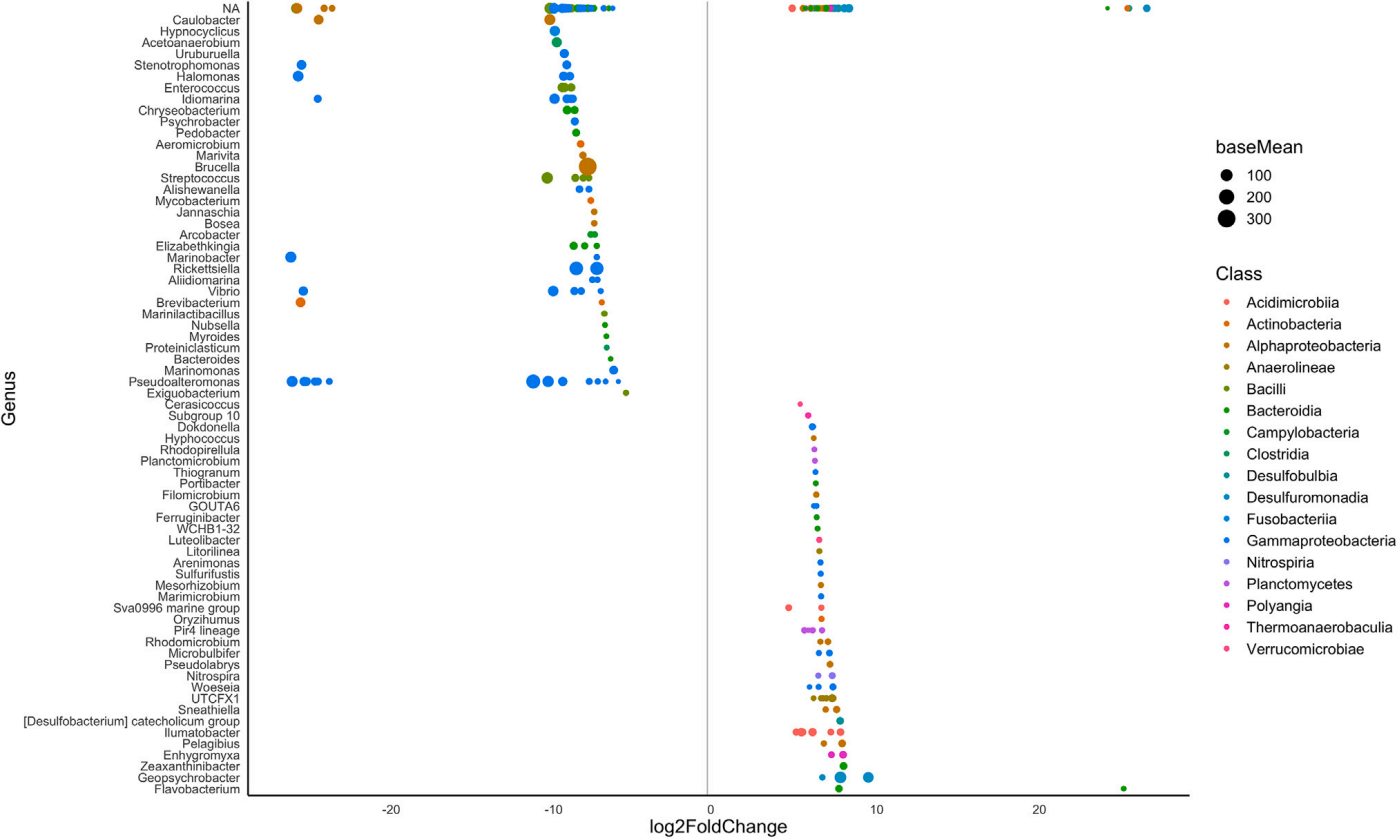
DESeq identified a total of 212 differentially abundant taxa between eastern Maine Nelson’s sparrow feather samples (n *=* 28) and sediment samples (n *=* 12) at a significance level of p *<* 0.01 The size of each symbol (baseMean in the legend) indicates the average of the normalized count values for each taxa. Taxa more abundant in feather samples are displayed on the left and those more abundant in sediment samples are displayed on the right.

Eastern Maine Nelson’s sparrow feather and eastern Maine sediment samples were inhabited by distinct bacterial communities (Figure 7). Feather samples, widely dispersed in the PCoA, demonstrate greater interindividual variability among samples than do the more tightly clustered sediment samples. Feather and sediment samples did not vary in group dispersion, and PERMANOVA results show a significant effect of sample type on community composition for both Jaccard and Bray-Curtis dissimilarity (*R*^*2*^ = 0.12, *F*_*1, 37*_ *=* 4.55, p *<* 0.001; *R*^*2*^ = 0.174, *F*_*1, 37*_ *=* 8.016, p *<* 0.001, respectively). Over 200 differentially abundant SVs were detected between feather and sediment samples (Figure 8). This same differentiation in community composition between sample types was also seen in the subset of potentially keratinolytic bacterial taxa (Jaccard dissimilarity: *R*^*2*^ = 0.11, *F*_*1, 34*_ *=* 4.206, p *<* 0.001; Bray-Curtis dissimilarity: *R*^*2*^ = 0.163, *F*_*1, 37*_ *=* 6.612, p *<* 0.001).

Fungal community composition varied significantly in homogeneity of variance between feather and sediment sample types for both Jaccard and Bray-Curtis dissimilarity (PERMUTEST, *F*_*1,79*_ = 36.04, p *=* 0.001; *F*_*1,79*_ = 11.12, p *=* 0.001, respectively). Feather communities harbored 14 fungal taxa at a higher abundance than sediment samples, and no sequence variants were found to be more abundant in sediment than feather samples (Table S13). In the subset of genera containing keratinolytic taxa, feather and sediment samples differed significantly in group dispersion under Jaccard (PERMUTEST, *F*_*1,65*_ = 4.73, p *=* 0.031) but not Bray-Curtis dissimilarity (PERMUTEST, *F*_*1,65*_ = 2.73, p *=* 0.11). Sample types did not differ in community composition under Bray-Curtis dissimilarity (PERMANOVA, *R*^*2*^ = 0.019, *F*_*1,65*_ *=* 1.25, p *=* 0.147).

#### Taxa shared between feather and sediment samples

**Figure 9:**
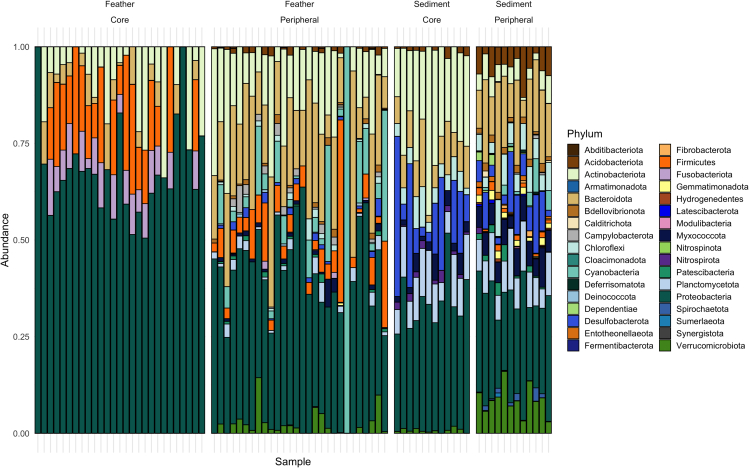
Relative abundance of bacterial phyla present in the core (defined as SVs present in >60% of samples) and peripheral (defined as SVs present in <20% of samples) communities of eastern Maine Nelson’s sparrow feather samples (n *=* 28) and eastern Maine sediment samples (n *=* 12)

Microorganism taxa comprising plumage and sediment core communities were identified and compared across sample types. Both plumage and sediment samples had small core bacterial communities, and taxa comprising these core communities differed between the sample types (Figure 9). Only eight SVs (0.3% of SVs) (Table 3) were found in >60% of eastern Maine Nelson’s feather samples, while 89 SVs (1.4%) occurred in >60% of eastern Maine sediment samples. Most bacterial taxa occurred in <20% of samples and were defined as peripheral microbiota in both feather (92.2% of SVs) and sediment (82.8% of SVs) communities. When using a >50% prevalence threshold for defining the core microbial community, 33 SVs (1.3%) were included in the feather core and 147 SVs (2.3%) were included in the sediment core community. In feather samples, 2,038 SVs (82.3% of total SVs) were found in <10% of samples and 4,098 SVs (65.8% of total SVs) were found in <10% of sediment samples.

**Table 3:** Taxonomy of bacterial SVs in >60% of eastern Maine Nelson’s sparrow feather samples (n *=* 28)

Phylum	Class	Order	Family	*Genus*	*Species*
Actinobacteriota	Actinobacteria	Propionibacteriales	Nocardioidaceae	*Nocardioides*	NA
Firmicutes	Bacilli	Bacillales	Bacillaceae	NA	NA
Firmicutes	Bacilli	Lactobacillales	Enterococcaceae	*Enterococcus*	NA
Fusobacteriota	Fusobacteriia	Fusobacteriales	Leptotrichiaceae	*Hypnocyclicus*	NA
Proteobacteria	Alphaproteobacteria	Caulobacterales	Caulobacteraceae	*Caulobacter*	NA
Proteobacteria	Alphaproteobacteria	Rhodobacterales	Rhodobacteraceae	NA	NA
Proteobacteria	Gammaproteobacteria	Enterobacterales	Pseudoalteromonadaceae	*Pseudoalteromonas*	NA
Proteobacteria	Gammaproteobacteria	Enterobacterales	Vibrionaceae	*Vibrio*	*parahaemolyticus*

For fungi, neither feather nor sediment samples had SVs that were present in >50% of unrarefied samples at a 0.1% detection threshold. Both sample types had large peripheral communities. In feather samples 7371 SVs (99.7% of total SVs) were detected in <20% of samples and 7254 SVs (98.1% of total SVs) were detected in <10% of samples. In sediment samples 1810 SVs (98.2% of total SVs) were found in <20% of samples and 1572 SVs (85.2% of total SVs) were found in <10% of samples.

In total, 564 bacterial SVs were shared between Nelson’s feather and sediment samples collected in eastern Maine (no prevalence or detection thresholds used). This represents only 7% of the SVs present across both sample types. Of these 564 shared SVs, 313 (55.5%) are present in <20% of both feather and sediment samples and were therefore defined as peripheral taxa in both sample types. An additional 201 SVs (35.6% of all shared taxa) shared between both sample types were defined as peripheral taxa in feathers (present in <20% of samples) but were more prevalent across sediment samples.

A small number of fungal SVs (610; 7%) were shared between feather and sediment samples. Most fungal SVs were found exclusively in feather samples (6,782; 79%). Only 1,233 (14%) of detected SVs were unique to sediment samples.

## Discussion

This study provides the first description of tidal marsh sparrow plumage microorganism communities, laying the groundwork for future investigations into coevolution between microbiota and tidal marsh sparrows. In comparison to plumage microorganism studies on other wild bird species, we report comparable alpha diversity and shared dominant bacterial phyla.^11^^,^^28^^,^^29^^,^^30^ Nelson’s sparrows had higher alpha diversity and distinct community composition as compared to saltmarsh, but not seaside, sparrows. However, the differentiation of Nelson’s and saltmarsh bacterial communities was observed when considering presence-absence, and not relative abundance, of taxa. The plumage bacterial communities of Nelson’s sparrows possessed a core community that was consistently dissimilar to sediment communities, and fungal taxa were more common and diverse in plumage relative to sediment samples. The results indicating that tidal marsh sparrows possess plumage microorganism communities that are distinct from those of sediment and include taxa commonly found on other wild avian hosts suggests that integument-microorganism coevolution may be possible. However, these findings do not support our hypothesis that sediment is a primary source of microorganism acquisition (and particularly of keratinolytic microorganisms). A high degree of inter-individual microorganism community variation in Nelson’s sparrow plumage samples suggests that these communities experience rapid turnover of peripheral taxa and the possibility that community members are acquired from multiple environmental and/or social sources. Collectively, this study highlights the complexity of wild microorganism communities and contributes to our growing understanding of the interplay between microorganisms, the environment, and host phenotype evolution, yet should be interpreted with caution given limited sample sizes.

### High bacterial and fungal diversity across all plumage samples

The tidal marsh sparrow plumage samples included in this study had similar bacterial diversity relative to previous avian microorganism studies. Compared to the 22 phyla we report in our study, 15 phyla were reported across sympatric skylark (*Lullula arborea*) and woodlark (*Alauda arvensis*) integument and nest material samples,^29^ and 22 phyla were reported in plumage samples from five species of sympatric Cathartiformes vultures.^30^ Four phyla dominant in this study’s plumage samples – Proteobacteria, Bacteroidota, Actinobacteria, and Firmicutes – are among the dominant phyla in several metabarcoding plumage bacteria studies.^11^^,^^28^^,^^29^^,^^30^ The total number of taxa present in our rarefied feather dataset (1,777 SVs) is lower than some studies (∼2,500 taxa in single plumage patches^11^^,^^30^), but comparable to those found in lark plumage (∼1,100^29^) and wild zebra finch (*Taeniopygia guttata)* skin swabs (∼1,200^31^). Such variation could be due to biological variation or differences in sample collection and molecular methods.^32^

To our knowledge, this study is the first to have performed metabarcoding of wild plumage fungal communities. Much of the metabarcoding work conducted on integumentary fungal microbial communities in natural systems has focused on reptiles and amphibians impacted by fungal pathogens.^33^^,^^34^ Previous studies of fungal communities associated with avian plumage have focused on the subset of fungal taxa that are culturable^35^^,^^36^^,^^37^^,^^38^ or have analyzed abundance (and not taxonomy) of fungal microorganisms.^39^ The most common phyla detected in our study, Ascomycota and Basidiomycota, were also abundant in studies on the integument of birds, reptiles, amphibians, and healthy humans.^33^^,^^34^^,^^36^^,^^40^ This study detected a substantially higher diversity of fungal taxa in plumage samples than a study of captive Quaker parrots (*Myiopsitta monachus*), which identified 97 fungal taxa as compared to the 5,125 described here.^41^ Fungal taxa were more diverse than bacterial taxa in our plumage samples. Conversely, sediment samples contained a higher number of bacterial than fungal taxa.

### Host species-specific bacterial communities

Bacterial, but not fungal, diversity and community composition were found to vary somewhat across sparrow host species (Figures 4 and 5). We discuss the potential implications of these results below but note that sample sizes are low for these between-species comparisons. Additional study will be needed to further elucidate patterns and their potential drivers.

Microorganism community composition variation across vertebrate taxa can be due to differences in the external environment and/or in host physiology and behavior.^42^^,^^43^^,^^44^ While all occupy saltmarsh habitats, Nelson’s, saltmarsh, and seaside sparrows have different geographic ranges (with some overlap) and habitat preferences. Nelson’s sparrows occur furthest north and often are found in small, upriver marshes with lower salinity and high plant diversity.^45^ Unlike the seaside and saltmarsh sparrows, Nelson’s sparrows are also known to make use of non-marsh habitats such as forest edges and fields,^46^ factors which may increase the diversity of environmental microbes to which Nelson’s sparrows are exposed. Seaside sparrows were not found to differ from Nelson’s or saltmarsh sparrows in alpha diversity or community composition but also had the lowest sample size of the three species. We therefore cannot conclude that seaside sparrows do not harbor distinct microorganism communities. Seaside sparrows exhibit unique behaviors – this species tends to nest and forage in low-marsh habitats, whereas Nelson’s and saltmarsh sparrows primarily use areas of high-marsh – and might be expected to therefore acquire a different microbial composition through association with the low-marsh microhabitat. Future work to increase host species sample sizes and perform a thorough survey of environmental microorganism communities across high and low marsh habitat types would help to elucidate if there are associations between sparrow species microorganism communities and microhabitat use. It is also possible that the variation detected across host species is due to differences in microorganism communities across marshes rather than those at the within-marsh microhabitat scale. The relatively weak differentiation in host species microorganism communities reported here is reflective of a broader trend in the literature where host species differentiation is not consistently detected across studies.^28^^,^^29^^,^^39^

Sequence variants belonging to three bacterial genera were found to be differentially abundant across the host species: *Rickettsiella, Marinobacter*, and *Pseudoalteromonas* (Table 2). One sequence variant within the genus *Rickettsiella* was found to be most abundant in saltmarsh sparrows and least abundant in Nelson’s sparrows. Arthropods are vectors of *Rickettsiella* pathogens, which can infect avian species.^47^ It is possible that variation in the abundance of the *Rickettsiella* SV is reflective of variation in tick prevalence across marshes. The genera *Marinobacter* and *Pseudoalteromonas* are both associated with marine habitats, and their differential abundance across the host species may be related to environmental differences across marshes.

Several fungal sequence variants were found to be differentially abundant across host species, despite a lack of significant variation in overall community composition. Four fungal genera – *Paraphaeosphaeri*, *Phaeosphaeria*, *Alternaria*, and *Penicillium –* with differentially abundant SVs across host species have been detected in association with saltmarsh vegetation.^26^^,^^48^ Both *Alternaria* and *Penicillium* contain taxa with keratinolytic and anti-microbial functions.^49^^,^^50^^,^^51^ Variation in fungal taxa abundance across host species likely reflects environmental variation across marshes, which may result in differential exposure of the host species to keratinolytic and/or anti-microbial fungi.

### Differentially abundant taxa despite minimal within-species plumage microorganism community variation

Within Nelson’s sparrow plumage samples, no variable of interest was found to have a significant influence on overall alpha diversity or community composition. However, it is possible that patterns in microorganism community variation may be revealed by future studies with higher sample sizes. Despite the lack of detected variation in alpha diversity and community composition, DESeq uncovered several differentially abundant microorganisms between certain sample groups (Table S11). These differentially abundant taxa suggest that certain plumage bacterial and fungal taxa may vary with environmental conditions among marshes, months, tidal cycles, and tidal ranges. Many of these bacterial taxa, such as SVs within the genera *Halomonas* and *Pseudoalteromona*, are halotolerant and commonly found in marine environments.^52^^,^^53^ Fungal taxa that varied among groups were frequently plant-associated endophytes, some of which – including *Phaeosphaeria halima,* taxa within the genera *Papiliotrema* and *Dioszegia*, and taxa within the family Neodevriesiaceae *–* have previously been found to cycle in abundance with the decay of saltmarsh vegetation.^54^ The association of many of these organisms with marine environments and vegetation indicates that their fluctuation amongst groups of feather samples is likely reflective of their abundance in the surrounding environment. The bacterial genus *Enterococcus* and the fungal genera *Alternaria* and *Cladosporium* contain keratinolytic taxa,^25^^,^^49^ indicating that exposure of tidal marsh sparrows to potentially antagonistic microorganisms may shift with environmental conditions. Of note, while no differentially abundant taxa were detected between female and male Nelson’s sparrows, female sparrows were found to harbor 14 fungal sequence variants at a higher abundance than males. Many of these taxa – *Symmetrospora symmetrica, Erythrobasidium hasegawianum, Papiliotrema taeanensis, Genolevuria* sp*.,* and *Dioszegia* sp. – are yeasts found in association with plants. The higher abundance of these 14 taxa in female Nelson’s sparrows may be the result of the increased exposure that females have to saltmarsh vegetation while building and incubating nests. While not assessed in this study due to limited sample size, exposure of females to specific microhabitats within the saltmarsh may vary with breeding stage (nest building, egg incubation, chick provisioning, etc.) and therefore potentially lead to cyclical shifts in microorganism communities (tidal marsh sparrows re-nest throughout the summer^55^). Future study of microorganism variation across breeding stages, as well as a broader investigation into the nest-specific microorganism community, may help in understanding the transfer of microorganisms between females, offspring, and the nest environment.

### Keratinolytic, pathogenic, and extremophile microorganism genera detected in tidal marsh sparrow plumage

Sequence variants belonging to bacterial genera containing keratinolytic taxa were detected in all 39 plumage samples, totaling to 91 SVs (4.1% of all SVs) from 12 bacterial genera across the samples (Table 4). These 12 bacterial genera are metabolically diverse, often associated with vertebrates, and some (such as *Kocuria* and *Janthinobacterium*) exhibit phenotypes that reflect adaptation to UV exposure, desiccation, and salinity,^56^^,^^57^ conditions likely reflective of the harsh conditions experienced by bacteria inhabiting plumage.

**Table 4:** Taxonomy of bacterial and fungal genera containing keratinolytic taxa detected the plumage of Nelson’s sparrows, saltmarsh sparrows, and seaside sparrows

Phylum	Class	Order	Family	*Genus*	*Species*	Number SVs
**Bacteria**						

Actinobacteriota	Actinobacteria	Micrococcales	Microbacteriaeae	*Microbacterium*	NA	4
Actinobacteriota	Actinobacteria	Micrococcales	Microbacteriaeae	*Kocuria*	NA	4
Bacteroidota	Bacteroidia	Flavobacteriales	Flavobacteriaceae	*Flavobacterium*	*rivuli*	1
Bacteroidota	Bacteroidia	Flavobacteriales	Flavobacteriaceae	*Flavobacterium*	*succinicans*	1
Bacteroidota	Bacteroidia	Flavobacteriales	Flavobacteriaceae	*Flavobacterium*	NA	17
Bacteroidota	Bacteroidia	Flavobacteriales	Weeksellaceae	*Chryseobacterium*	NA	8
Firmicutes	Bacilli	Brevibacillales	Brevibacillaecae	*Brevibacillus*	NA	5
Firmicutes	Bacilli	Lactobacillales	Enterococcaceae	*Enterococcus*	NA	7
Firmicutes	Bacilli	Paenibacillales	Paenibacillaceae	*Paenibacillus*	NA	3
Firmicutes	Bacilli	Staphylococcales	Staphylococcaceae	*Staphylococcus*	*auricularis*	1
Firmicutes	Bacilli	Staphylococcales	Staphylococcaceae	*Staphylococcus*	NA	7
Proteobacteria	Gammaproteobacteria	Burkholderiales	Alcaligenaceae	*Acaligenes*	NA	4
Proteobacteria	Gammaproteobacteria	Burkholderiales	Alcaligenaceae	*Acaligenes*	endophyticus	1
Proteobacteria	Gammaproteobacteria	Burkholderiales	Oxalobacteraceae	*Janthinobacterium*	NA	1
Proteobacteria	Gammaproteobacteria	Pseudomonadales	Pseudomonadaceae	*Pseudomonas*	*flourescens*	1
Proteobacteria	Gammaproteobacteria	Pseudomonadales	Pseudomonadaceae	*Pseudomonas*	NA	13
Proteobacteria	Gammaproteobacteria	Xanthomonadales	Xanthomonadaceae	*Stenotrophomonas*	*maltophilia*	1
Proteobacteria	Gammaproteobacteria	Xanthomonadales	Xanthomonadaceae	*Stenotrophomonas*	NA	8
Proteobacteria	Gammaproteobacteria	Enterobacterales	Yersiniaceae	*Serratia*	NA	4
						Total: 91

**Fungi**						

Ascomycota	Dothideomycetes	Capnodiales	Cladosporiaceae	*Cladosporium*	*halotolerans*	1
Ascomycota	Dothideomycetes	Capnodiales	Cladosporiaceae	*Cladosporium*	NA	1
Ascomycota	Dothideomycetes	Capnodiales	Mycosphaerellaceae	*Acrodontium*	NA	1
Ascomycota	Dothideomycetes	Pleosporales	Phaeosphaeriaceae	*Neosetophoma*	NA	3
Ascomycota	Dothideomycetes	Pleosporales	Pleosporaceae	*Alternaria*	*betae-kenyensis*	1
Ascomycota	Dothideomycetes	Pleosporales	Pleosporaceae	*Alternaria*	*metachromatica*	1
Ascomycota	Dothideomycetes	Pleosporales	Pleosporaceae	*Alternaria*	*molesta*	1
Ascomycota	Dothideomycetes	Pleosporales	Pleosporaceae	*Alternaria*	NA	44
Ascomycota	Dothideomycetes	Pleosporales	Pleosporaceae	*Alternaria*	*rosae*	1
Ascomycota	Dothideomycetes	Pleosporales	Pleosporaceae	*Curvularia*	*intermedia*	1
Ascomycota	Dothideomycetes	Pleosporales	Pleosporaceae	*Curvularia*	NA	2
Ascomycota	Eurotiomycetes	Eurotiales	Aspergillaceae	*Aspergillus*	*halophilicus*	1
Ascomycota	Eurotiomycetes	Eurotiales	Aspergillaceae	*Aspergillus*	NA	2
Ascomycota	Eurotiomycetes	Eurotiales	Aspergillaceae	*Aspergillus*	*penicillioides*	1
Ascomycota	Eurotiomycetes	Eurotiales	Aspergillaceae	*Penicillium*	*bialowiezense*	1
Ascomycota	Eurotiomycetes	Eurotiales	Aspergillaceae	*Penicillium*	NA	10
Ascomycota	Eurotiomycetes	Eurotiales	Aspergillaceae	*Penicillium*	*oxalicum*	1
Ascomycota	Eurotiomycetes	Eurotiales	Aspergillaceae	*Penicillium*	*spinulosum*	1
Ascomycota	Eurotiomycetes	Eurotiales	Aspergillaceae	*Penicillium*	*xanthomelinii*	1
Ascomycota	Eurotiomycetes	Eurotiales	Trichocomaceae	*Talaromyces*	*helicus*	1
Ascomycota	Eurotiomycetes	Eurotiales	Trichocomaceae	*Talaromyces*	NA	5
Ascomycota	Eurotiomycetes	Eurotiales	Trichocomaceae	*Talaromyces*	*ramulosus*	2
Ascomycota	Leotiomycetes	Helotiales	Helotiales	*Cadophora*	*malorum*	1
Ascomycota	Leotiomycetes	Helotiales	Helotiales	*Cadophora*	*melinii*	1
Ascomycota	Leotiomycetes	Helotiales	Helotiales	*Cadophora*	NA	1
Ascomycota	Leotiomycetes	Helotiales	Myxotrichaceae	*Oidiodendron*	*maius*	1
Ascomycota	Leotiomycetes	Helotiales	Myxotrichaceae	*Oidiodendron*	NA	2
Ascomycota	Saccharomycetes	Saccharomycetales	Saccharomycetales	*Candida*	*argentea*	3
Ascomycota	Saccharomycetes	Saccharomycetales	Saccharomycetales	*Candida*	NA	3
Ascomycota	Saccharomycetes	Saccharomycetales	Saccharomycetales	*Candida*	*sake*	10
Ascomycota	Saccharomycetes	Saccharomycetales	Saccharomycetales	*Candida*	*spencermartinsiae*	1
Ascomycota	Saccharomycetes	Saccharomycetales	Saccharomycetales	*Candida*	*tropicalis*	1
Ascomycota	Sordariomycetes	Hypocreales	Cordycipitaceae	*Engyodontium*	*album*	1
Ascomycota	Sordariomycetes	Hypocreales	Hypocreaceae	*Trichoderma*	*asperellum*	1
Ascomycota	Sordariomycetes	Hypocreales	Hypocreaceae	*Trichoderma*	NA	1
Ascomycota	Sordariomycetes	Hypocreales	Nectriaceae	*Fusarium*	*concentricum*	1
Ascomycota	Sordariomycetes	Hypocreales	Nectriaceae	*Fusarium*	NA	23
Ascomycota	Sordariomycetes	Hypocreales	Nectriaceae	*Fusarium*	NA	2
Ascomycota	Sordariomycetes	Hypocreales	Stachybotryaceae	*Myrothecium*	*gramineum*	1
Ascomycota	Sordariomycetes	Hypocreales	Stachybotryaceae	*Myrothecium*	NA	2
Ascomycota	Sordariomycetes	Hypocreales	Stachybotryaceae	*Stachybotrys*	*aloeticola*	2
Ascomycota	Sordariomycetes	Hypocreales	Stachybotryaceae	*Stachybotrys*	NA	1
						Total: 160

Some taxa within the 12 detected genera are known host antagonists^58^^,^^59^ and many have been studied for their ability to degrade feathers from poultry waste (citations in Table S1). However, some of these producers of keratinase, such as *Pseudomonas*, can also produce bacteriocins,^60^^,^^61^ and may therefore help support microbial communities that are commensal with feathers.^28^ Such interactions between microbial taxa and host integument have been documented in amphibians, which experience lower *Batrachochytrium dendrobatidis*-induced mortality rates when inoculated with *Janthinobacterium lividum*.^62^ This bacterium produces the compound violacein, which confers resistance to environmental stressors and has anti-fungal properties. Functional analyses will be required to understand how these taxa interact with other microorganisms and their sparrow hosts.

Contrary to expectations, despite uncovering nearly 90 SVs belonging to genera containing keratinolytic bacteria among our feather samples, the often-studied *Bacillus licheniformis* was not detected. This discrepancy may be the result of biases introduced by choice of laboratory methods. Many prior studies of plumage microbial communities have used culturing techniques that specifically select for or are more likely to discover high abundances of *Bacillus* than culture-independent methods.^63^ Due to their thick cell walls, gram-positive bacteria tend to be more difficult to lyse for DNA extraction,^64^ potentially biasing molecular methods against the detection of *Bacillus.* However, members of the genus *Bacillus* were detected at low levels in our unrarefied sediment samples, indicating that our molecular techniques could detect these taxa. Given that a culture-based study revealed high abundances of *Bacillus* in the plumage of the coastal plains swamp sparrow,^19^ comparing culture and molecular-based techniques on the same set of tidal marsh feather and sediment samples would aid in determining if this study’s lack of *Bacillus* is due to methodological or biological conditions. Regardless, this study’s discovery of a wide diversity of potentially keratin-degrading taxa highlights the importance of studying entire microorganism communities, moving beyond focusing on individual taxa.

Fungal genera containing keratinolytic taxa were present in 58 plumage samples (90.8% of samples) and included a total of 160 SVs (3.1% of all SVs). These SVs belonged to 16 genera that have each been associated with degradation of poultry feathers (summarized in Table S2). The most speciose of these genera were *Alternaria* with 51 SVs and *Fusarium* with 24 SVs. Fungi classified as *Alternaria* or *Fusarium* produce a broad range of metabolites and can act as saprophytes, plant endophytes, and/or plant pathogens.^65^^,^^66^ Many of the fungal genera detected in plumage samples, including *Alternaria, Fusarium, Cladosporium, Candida, Penicillium, Aspergillus, Curvularia, Trichoderma,* and *Talaromyces*, produce secondary metabolites with antimicrobial activity.^51^^,^^67^^,^^68^ Species in the genus *Aspergillus*, while detected in low abundance in this study (five *Aspergillus* SVs were detected in low abundance in six plumage samples) are known to cause respiratory infection that can lead to mortality in avian hosts.^69^ Similar to the subset of bacterial genera with known keratin-degraders, the metabolic diversity of keratinolytic fungal taxa indicates that these fungi may exhibit a variety of interactions with feathers and other microorganisms in the community. However, these taxa were found in similar abundance in sediment samples. This result indicates a potential lack of specialization to the feather environment, and the possibility that these taxa are dormant spores that are not interacting directly with feathers.

### Specialized, but highly variable plumage microorganism communities

Overall, Nelson’s sparrow plumage and Maine sediment bacterial communities differed markedly in alpha diversity and community composition. These observations indicate that, despite frequently coming into contact with marsh sediments, plumage bacterial communities are not simply a subset of taxa acquired from sediment. Further, very few bacterial or fungal taxa are shared between feather and sediment communities, and most SVs that are shared between the sample types occur in only a small percentage of samples. This distinction between plumage and sediment microorganism communities, a pattern observed in previous research,^70^ may be driven by acquisition of taxa from other environmental or social sources, and/or selection of environmental microorganisms by conditions specific to the host.^71^^,^^72^ Additional sources of environmental acquisition may include unsampled sediments (such as at nesting or foraging locations), marsh vegetation (namely grasses and sedges), and water, which exists at a range of salinities across the marsh and during the daily and monthly tidal cycles. Sources of social acquisition may include transmission of microbes to nestlings from female integument and the nesting environment,^73^ or from contact between adult individuals^31^ during antagonistic or copulatory events, which are both frequent in this system.^74^

Once obtained, certain microorganisms may be selected for by the environment of the feather, which provides nutrients in compounds such as preen oils and keratin but is frequently exposed to salinity and UV radiation. Reflective of the harsh feather environment, the bacterial genera comprising the Nelson’s sparrow plumage core community are adapted to extreme environments: *Nocardioides* is associated with hypersaline lakes;^75^ *Hypnocyclius* has been isolated from marine hydrothermal vents;^76^ and *Caulobacter, Vibrio*, *Pseudoalteromonas*, and Rhodobacteraceae are often found in saline waters.^53^^,^^77^^,^^78^^,^^79^^,^^80^ Potential specialization to and interaction with the integumentary environment is seen in the dominant phyla of the feather bacteria community, all of which are frequently found in association with avian feathers.^11^^,^^28^^,^^29^^,^^30^ Also indicative of specialization to the integument, many of the genera containing keratinolytic taxa detected in this study are often isolated from avian plumage or sources of poultry waste.^2^^,^^41^^,^^63^^,^^81^ Several of the taxa detected in the core feather bacterial community and in the genera containing keratinolytic taxa may interact commensally with feathers by regulating microbes; *Caulobacter*, *Pseudoalteromonas*, *Bacillus*, and *Pseudomonas* each have antimicrobial activity.^60^^,^^61^^,^^79^^,^^82^

While these results indicate that certain microbes may be specifically adapted to vertebrate hosts, it appears that many taxa present within a given feather sample are part of a peripheral community of microorganisms that do not establish consistent populations across the feather environment of many individual birds. Eastern Maine Nelson’s sparrow plumage samples exhibited substantial inter-individual variation that remains unexplained by our investigated variables of feather color, sex, marsh, month in the breeding season, tidal cycle, and tidal range. These samples demonstrate high dissimilarity between samples from recaptured individuals (Table S3), a wide scattering of samples on PCoA (Figure S2), a small bacterial core community, and a non-existent fungal core community. Given this high level of unexplained variation, we hypothesize that the microorganism communities that we describe from each sample represent a snapshot of diversity that may relate to unmeasured environmental variation and an individual bird’s most recent behaviors and movements throughout the marsh. For example, a microbial community dominated by Cyanobacteria (as seen in one of our Nelson’s sparrow plumage samples) may reflect a recent bath in a Cyanobacteria-dominated pool or panne just prior to sampling. Birds are also known dispersers of fungal spores that attach to feathers,^83^ suggesting that it is common for plumage to harbor inert environmental fungi. These initial findings warrant further investigation into the stability of tidal marsh sparrow core and peripheral plumage microorganism communities over time.

### Plumage microorganisms and saltmarsh melanism – An area for future study

If coevolution between vertebrate hosts and their microorganism communities has occurred, we would expect to see common phenotypic responses across hosts that are inhabited by similar communities. Such is the case with tidal marsh sparrows, which all exhibit a suite of adaptations to tidal marsh environments that includes increased plumage melanism, a pattern termed saltmarsh melanism.^84^ Like other saltmarsh-specific traits, saltmarsh melanism is least pronounced in Nelson’s sparrows, the species with the shortest evolutionary history in the habitat. It has been hypothesized that saltmarsh melanism evolved as a defense against bacteria^19^ – melanin pigments have anti-microbial properties,^85^ which may play a role in regulating plumage microbial communities,^86^^,^^87^ and increased pigmentation of feathers has been correlated with higher resistance to both physical and microbial degradation.^88^^,^^89^^,^^90^^,^^91^

This study has established that the conditions exist for potential coevolution between tidal marsh sparrows and microorganism communities, and such coevolution could be further studied for a relationship with saltmarsh melanism. Our results demonstrate that the microorganism communities of sparrow plumage are composed of bacterial and fungal phyla that have been detected in the integument of other avian species and contain taxa that may have pathogenic, anti-microbial, and/or keratinolytic functions. Further, bacterial (but not fungal) communities are distinct from those of saltmarsh sediments. While results of this study demonstrate ecological (and not co-evolutionary) associations between sparrow species and microorganisms, the functionally diverse, vertebrate-specific taxa we describe here could be candidates for further investigation for their potential to exert selective pressures upon hosts.

### Conclusions

This study serves as a first step toward future work on potential coevolutionary dynamics between tidal marsh sparrow plumage phenotypes and microorganisms. Bacterial alpha diversity and community composition varied between Nelson’s and saltmarsh sparrows, a pattern which may be driven by differences in within-marsh habitat use or environmental differences across marsh habitats. Differences in abundance of several microorganism taxa in Nelson’s sparrow plumage samples appear to be due to environmental, temporal, and sex-specific microorganism variation. Bacterial and fungal genera that contain keratinolytic taxa were found across plumage samples but only comprised a small percentage of the plumage microorganism community. Functional studies (such as metagenomics and/or metabolomics) are necessary to confirm if and how these taxa interact with avian hosts and other plumage microorganisms. Feather and sediment bacterial communities were found to be distinct, indicating that the sediment is not the primary source of bacterial acquisition for ground-foraging tidal marsh sparrows and that these birds likely host taxa from multiple environmental sources, as well as some vertebrate-specialized taxa. However, plumage and sediment fungal communities were similar in alpha diversity and community composition, reflecting the potential acquisition of dormant fungal spores from the environment. Finally, we hypothesize that the high degree of inter-individual variation in plumage microorganism communities is generated by the presence of a large, rapidly shifting community of peripheral taxa that are not necessarily adapted to conditions of the integument and therefore exhibit frequent turnover. Collectively, these findings demonstrate the multiplicity of host and environmental drivers of microorganism community variation and suggest that tidal marsh sparrows harbor (1) microorganisms (especially bacteria) that are vertebrate and/or sparrow species-specific and candidates for future studies of host-microbe coevolution and (2) transient taxa that are reflective of unique environments and behaviors but are less likely to have influenced tidal marsh sparrow phenotypes.

### Limitations of the study

Integumentary structures have low microorganism biomass, posing challenges for molecular analysis.^92^ Further, amplifying DNA from saline sediments can be difficult as salts and other impurities inhibit amplification.^93^ As a result, feather and sediment samples had highly variable sequencing depths and samples with <1,000 reads were subsequently removed from analysis. We therefore achieved small sample sizes of sequencing data despite extensive field collections, which may reduce our statistical power to detect fine-scale differences among sample groups. The interpretation of our findings is also limited to microorganism identity due to the use of metabarcoding techniques. While we can infer function based on connections to the known functions of related taxa, a metagenomics study would be necessary to confirm the functional importance of the communities described in this study.

## STAR★Methods

### Key resources table

**Table:** 

REAGENT or RESOURCE	SOURCE	IDENTIFIER
**Critical commercial assays**		

QiaAmp DNA Micro Kit	Qiagen	Cat. no.: 56304
QIAquick PCR	Qiagen	Cat. no.: 28104

**Deposited data**		

Raw data	This paper	SRA: PRJNA925448
Code for final analyses	This paper	https://github.com/alicehotopp/TidalMarshSparrow-Microorganisms.git

**Oligonucleotides**		

ITS Primers 5.8S-Fun: AACTTTYRRCAAYGGATCWCT ITS4-Fun: AGCCTCCGCTTATTGATATGCTTAART	Taylor et al.^94^	N/A
16S Primers Bakt_341: CCTACGGGNGGCWGCAG Bakt_805: GACTACHVGGGTATCTAATCC	Klindworth et al.^95^	N/A

**Software and algorithms**		

R/R Studio	R Core Team 2022	https://www.r-project.org/

### Resource availability

#### Lead contact

• Further requests for information or resources should be directed to and will be fulfilled by the lead contact, Alice Hotopp (alice.hotopp@maine.edu)

#### Materials availability

• This study did not generate new unique reagents.

#### Data and code availability

• FASTA files have been deposited at the NCBI Short Read Archive and are publicly available as of the date of publication. Accession numbers are listed in the key resources table.
• All original code has been deposited at GitHub and is publicly available as of the date of publication. DOIs are listed in the key resources table.
• Any additional information required to reanalyze the data reported in this paper is available from the lead contact upon request.

### Experimental model and study participant details

This study field-captured and sampled plumage from tidal marsh sparrows, which were released after sampling. Plumage samples were taken from 38 Nelson’s sparrows (10 females, 28 males), 20 saltmarsh sparrows (4 females, 16 males), and 11 seaside sparrows (3 females, 8 males) from coastal sites from Maine to New Jersey in the United States (Figure 2; Table 1). Only adult birds were sampled in this study. Information on the sex and geographic site of sampled birds can be found in metadata files available on Short Read Archive (accession numbers in the key resources table). The study was approved by Ethical committee No. 272/MSAS/DPRS/CNRS 28 May 2014 and informed consent was ensured. Appropriate animal care was ensured by the Institutional Animal Care and Use Committee of the University of Maine under approval A2019-04-02, University of New Hampshire under approval 22041, and University of Connecticut under approval A21-008. Capture, banding, and feather sample collection were conducted under Maine state permits #2021-270 and #2021-314, Connecticut state permit #0226012, New Jersey state permit #SC 2020109, and US Federal permits #23613, and #24045. Work at Edwin B. Forsythe NWR was conducted under Special Use Permit #2021-013.

### Method details

#### Methods

##### Study system: Brief overview of saltmarsh habitats

Tidal saltmarshes, common along North America’s Atlantic coast, are ecotones that experience cyclical tidal flooding and desiccation. These habitats have zonation of vegetation based on frequency of daily inundation – low marsh zones exist below the mean high-water mark and flood with the daily high tide, whereas high marsh zones exist above this mark.^96^ Saltmarshes are also affected by monthly tidal dynamics that are driven by the lunar cycle. Spring tides occur during full or new moons (when the gravitational pull upon oceans is heightened with the earth, sun, and moon in alignment) and lead to extreme high and low tides.^97^ Near-complete flooding of marshes often occurs during spring tides. Neap tides are moderate and occur in the period between spring tides.

##### Sampling scheme by research aim

*Aim 1: Interspecific microorganism community variation –* To determine if host species have distinct microbial communities, plumage samples were collected from Nelson’s, saltmarsh, and seaside sparrows from marshes in four regions: Washington County, Maine (Nelson’s sparrow: *n = 22*); Rachel Carson Wildlife Refuge, Wells, Maine (Nelson’s sparrow: *n* = 8; saltmarsh sparrow: *n =* 24); Hammonasset Beach State Park, Madison, Connecticut (saltmarsh sparrow: *n* = 28*;* seaside sparrow: *n* = 8); and Edwin B. Forsythe National Wildlife Refuge, Galloway, New Jersey (saltmarsh sparrow: *n* = 22; seaside sparrow, *n* = 30) (Figure 2A). This collection effort was conducted during the June spring tide to minimize temporal and tide-cycle variation across sampling locations and species.

*Aim 2: Intraspecific microorganism community variation –* To explore microbial community variation within one species and one general geographic area, plumage samples (*n* = 68) were collected from Nelson’s sparrows across four distinct marshes in Washington County in the eastern region of Maine (Narraguagus River, Milbridge; Beaver Meadow Brook, Milbridge; Harrington River, Harrington; Pleasant River, Addison; Figure 2B) from the June spring tide until late August, the end of the breeding season. Variables investigated for their influence on microbial communities included feather color (increased pigmentation of feathers has been linked to microorganism regulation^87^), sex, marsh, month in the breeding season, tidal cycle (characterized based on the lunar cycle as spring or neap), and tidal range (difference in meters between low and high tide).

*Aim 3: Comparison of plumage and sediment microorganism communities –* To explore saltmarsh sediment as a source of microorganism acquisition, composite sediment samples (*n* = 34) were collected from the net location during each netting effort (for both Aims 1 & 2). Mist nets were set up in areas of the marsh with the highest bird densities to maximize the number of birds captured. Tidal marsh sparrows often travel by foot underneath vegetation. Captured individuals could therefore be reasonably expected to have been exposed to the sediment near the net location. Because of this centrality to bird presence in the marsh, the net site was chosen as the sediment sampling location. However, centering our sampling at mist net sites may have inadvertently increased the chances that sediments were disturbed by technicians while setting up mist nets.

##### Field methods – Plumage and sediment sample collection

Birds were captured using mist-netting techniques. One to six 12 m mist-nets were strung in arrays and birds were periodically flushed towards the nets. Technicians sanitized their hands prior to extracting birds from mist nets, and feather samples were sampled immediately after returning with the bird to the banding station. To minimize cross-contamination between individuals, birds were carried in single-use bags. Two samples of three feathers each were collected from each captured bird. Three feathers were plucked from both the darkly streaked upper region of the breast and from the region directly inferior, which is a uniformly white plumage patch (Figure S1). These two regions were sampled to assess if feather color (specifically, increased melanization of the upper breast feathers in comparison to the nearby lighter-colored lower breast feathers) is associated with a distinct microbial community. It is possible that cross-contamination of the upper and lower breast feathers occurred during the time birds spent in the paper bags (between 1 – 10 minutes) after extraction and prior to sampling. However, feathers samples were collected after extraction from mist nets to reduce exposure of birds to heat and direct sunlight while in mist nets.

Composite samples of surface sediment were collected from the middle and both ends of the net array. All feather and sediment samples were stored in DNA/RNA shield and kept on dry ice or ice packs while in the field and transferred to a -80°C freezer as soon as possible.

##### Molecular lab methods – DNA extraction, amplification, and sequencing

DNA was extracted from feather and sediment samples via an initial enzymatic lysis step,^11^ followed by a protein digestion step and QiaAmp DNA Micro Kit (Qiagen) extraction. After thawing and removal of storage buffer, 850 μl of lysis buffer (20 mM Tris-HCL, 2 mM EDTA, 1.2% Triton X-100, 20 ng/ml lysozyme) was added to samples. Samples were then pulse-vortexed for 15 seconds and incubated in a 37°C water bath for 60-75 minutes. After incubation, 200 μl sterile distilled H_2_O was added to tubes to promote settling of feather particles, and samples were centrifuged at 11,000 rpm for 10 minutes to pellet bacterial cells. Removal of supernatant was followed by a protein digestion step which consisted of incubating samples in 2 μl Beta-Mercaptoethanol, 20 μl Proteinase-K, and 180 μl ATL (QiaAmp DNA Micro Kit buffers, Qiagen) overnight in a 56°C water bath. The manufacturer’s protocol for DNA isolation from tissues was then followed.

Samples from different marshes, collection dates, host species, and sample types were included in each extraction group to minimize batch effects. A negative kit control was extracted alongside the samples in each group (samples were extracted in 21 batches), and DNA yields were quantified using a Qubit 4 Fluorometer (Invitrogen). DNA yields from feather samples ranged from too low to be read to 2.41 ng/ul, with an average of 0.183 ng/ul. Sediment sample DNA yields ranged from too low to be read to 255 ng/ul, with an average of 26.5 ng/ul.

Two rounds of PCR were performed to 1) amplify target DNA regions and 2) attach dual-index sequences to the PCR products to allow for pooling of samples during sequencing. The 16S rRNA gene and fungal ITS region were amplified from both feather and sediment DNA extracts using the primers Bakt_341 and Bakt_805 (approximate amplicon size of 464 bp; Klindworth et al. 2013) and ITS4-Fun and 5.8S-Fun (approximate amplicon size of 440 bp; Taylor et al. 2016^94^), respectively.

For feather DNA extracts, PCR amplification of 16S rRNA and ITS regions was performed in a final volume of 24 μl with 1.4 μl of each primer (5 μM), 12 μl KAPA HiFi HotStart ReadyMix (KAPA Biosystems, Wilmington, MA, USA), 5.2 μl sterile distilled H_2_0, and 4 μl DNA extract. Trials demonstrated that amplification of sediment extracts performed better with lower concentrations of DNA extract. Accordingly, the bacterial 16S rRNA region from sediment DNA extracts was amplified with 2.5 μl DNA extract, while the fungal ITS region was amplified with 1μl DNA extract.

Thermocycler conditions for 16S rRNA amplification consisted of denaturation at 94°C for 3 min, followed by 25 cycles of denaturation (94°C for 30 s), annealing (50°C for 30s), and elongation (72°C for 30 s), and finished with a final elongation step of 72°C for 7 min (unpublished protocol, Hubbard Center for Genome Studies, Durham, NH, USA). Conditions for ITS amplification started with denaturation at 96°C for 2 min, followed by 27 cycles of denaturation (94°C for 30 s), annealing (58°C for 40 s), and elongation (72°C for 2 min), followed by a final elongation step of 72°C for 10 min.^98^

The dual-index PCR reactions consisted of 6 μl of 2x KAPA HiFi HotStart ReadyMix, 0.7 μl of each primer (5 μM), 6 μl sterile distilled H_2_O, and 1 μl of template for a total volume of 12 μl.^99^ Thermocycler conditions consisted of denaturation at 95°C for 3 min, followed by 12 cycles of denaturation (98°C for 30 s), annealing and extension combined (72°C for 15s), and finished with a final elongation step of 72°C for 5 min.^99^

Following PCR, 4 μl of each sample was pooled and cleaned using a QIAquick PCR purification kit (Qiagen). A Qubit fluorometer (Life Technologies) was used to estimate library concentration, and approximately 1.1 nM of product was loaded for 250bp paired-end sequencing on a NovaSeq 6000 using V1.5 chemistry. Samples were sequenced across a total of four sequencing runs.

### Quantification and statistical analysis

#### Data pre-processing

##### Sequence data processing

Both 16S rRNA and ITS FASTQ files from each sequencing run were separately processed using the DADA2 pipeline^100^ in RStudio^101^ (R version 4.2.1). The package *ShortRead*^102^ was used to assess sequence quality. To trim low-quality scores, the first 10 and last 35 base pairs of reads were removed from 16S rRNA sequences. Cutadapt^103^ was used to remove primer sequences from the ITS sequences. The forward reads of the 16S rRNA sequences consistently had higher quality than the reverse reads whereas both the forward and reverse reads of ITS were high quality. Therefore, only forward reads were used for downstream 16S rRNA analysis while merged forward and reverse reads were used for ITS. Retained sections of reads had an average Phred score of 35.3 for 16S rRNA and 32.4 for ITS. DADA2 was used to calculate error rates, identify sequence variants (SVs), and remove chimeric reads (number of reads lost at filtering and chimera-removal steps summarized in Table S14). Taxonomic resolution at the level of SV was chosen allow for comparisons across studies.^104^^,^^105^ The database SILVA 138.1^106^^,^^107^ was used to assign taxonomy to 16S rRNA sequences, and taxonomy was assigned to ITS sequences using UNITE 8.3.^108^^,^^109^ The package *dplyr*^110^ was used to remove sequences identified as eukaryotic mitochondria and chloroplast from the 16S rRNA sequences. Sequences from all runs were combined with taxonomy assignments and metadata into a single object using the *phyloseq* package,^111^ and sequence variants identified in extraction and PCR negative controls were subtracted from sample sequences. Within *phyloseq*, the dominant taxa in each sequencing run were plotted, allowing for identification of evidence of contamination by the phylum Cyanobacteria across the majority of 16S rRNA samples in the second sequencing run, which was removed entirely from subsequent analysis. No evidence of contamination was identified in the ITS sequences. Principal Coordinates Analysis (PCoA) with Jaccard dissimilarity was used to visually identify sequencing run batch effects (indicated by tight clustering of samples within a single sequencing run). Sequencing runs that did not demonstrate batch effects (runs 3 & 4 for 16S rRNA and runs 1 - 4 for ITS) were retained for downstream analysis.

##### Assessing pairwise dissimilarity: Recaptured birds, resequenced samples, and feather color

Sequencing data contained three subsets of samples that were suspected to have higher average pairwise similarity than other groups of samples, which would make them non-independent from one another. These were duplicate samples of different feather colors from the same bird (16S rRNA: *n* = 48*; ITS: n* = 106) as well as samples from recaptured individuals 16S rRNA: *n* = 6*; ITS: n* = 19*)* and resequenced samples (PCR products from the same feather sample that were sequenced in distinct sequencing runs; 16S rRNA: *n* = 6*; ITS: n* = 33). The similarity of microorganism communities within each subset of samples was compared to the average similarity between a random subset of independent samples to help determine if data from different feather colors, recaptured birds, and resequenced products should be merged to minimize comparisons made between non-independent samples.

Initial investigation suggested that microorganism communities varied little between dark and white breast feathers (Figures S3 and S4). To test if feather color influenced bacterial community composition within individuals, pairwise Jaccard and Bray-Curtis dissimilarity was calculated (package *vegan*^112^) between the dark and white feathers from each individual, among all dark feathers, and among all light feathers (to compare feathers of the same color across different individuals). From the dissimilarity matrix comparing all dark feathers to each other, 10 sets of randomly selected pairwise dissimilarities where each individual bird was represented once were created. This was repeated for the dissimilarity matrix comparing all white feathers to each other. Kruskal-Wallis tests were performed to assess variation in community composition between feather colors by comparing pairwise dissimilarities of A) dark feather samples to paired dark and white feather samples and B) white feather samples and paired dark and white feather samples for each of the 10 sets of pairwise dissimilarities. Bacterial communities inhabiting the dark and white feathers from the same individuals were nearly always more similar than samples of the same color from different individuals for both Jaccard (dark feathers: 10/10 tests; white feathers: 10/10 tests) and Bray-Curtis dissimilarity (dark feathers: 10/10 tests; white feathers: 9/10 tests). Fungal communities of paired feather samples from the same individuals were also frequently more similar than samples of the same color from different individuals for Jaccard (dark feathers: 8/10 tests; white feathers: 9/10 tests) and Bray-Curtis dissimilarity (dark feathers: 10/10 tests; white feathers: 10/10 tests). These results indicated that feather color did not have a significant effect on microorganism community composition within individuals. To further assess the effect of within-individual feather color on microorganism communities, a paired Wilcox test was performed to assess if observed richness (number of SVs) and Shannon diversity varied between feather colors. Neither metric varied significantly between feather colors for the same individuals in bacteria (*V* = 136, p *=* 0.69; *V =* 114, p *=* 0.32, respectively) or fungal communities (*V* = 761, p *=* 0.52, *V =* 744, p *=* 0.62, respectively). As a result of these findings, sequencing reads from the dark and white feathers were combined for each bird for all subsequent analyses.

Similarly, to determine if bacterial and fungal communities of samples from either A) the same individual during different capture events or B) multiple sequencing runs from the same PCR product were more similar to each other than to DNA sequences obtained from different birds, pairwise Jaccard dissimilarity was calculated within the groups of recaptured birds and resequenced samples as well as within the dataset of all birds. From the dissimilarity matrix comparing all birds, 10 sets of pairwise dissimilarities where each set consisted of individuals captured once were randomly selected. Each of these 10 sets of pairwise dissimilarities was compared to the recaptured and resequenced dissimilarities using Kruskal-Wallis tests.

For bacterial communities, the dissimilarity between the recaptured individuals was never significantly different from the dissimilarity between all individuals for both Jaccard and Bray-Curtis dissimilarity. Recaptured individuals infrequently had significantly lower fungal community pairwise dissimilarity (Jaccard: 1/10 tests; Bray-Curtis: 0/10). These results indicated that samples from recaptured birds are no more similar to each other than any random subsample of different individuals. Samples from the recaptured birds were therefore treated as independent samples for both bacteria and fungi. Pairwise dissimilarity between resequenced samples tended to be lower than pairwise dissimilarity between two randomly selected samples. Bacterial communities of resequenced samples had significantly lower pairwise dissimilarity in few Kruskal-Wallis tests (Jaccard: 1/10 tests; Bray-Curtis: 3/10 tests). However, resequenced sample fungal communities often had significantly lower pairwise dissimilarity (Jaccard: 8/10 tests; Bray-Curtis: 8/10), indicating higher similarity between resequenced samples than independent samples. Therefore, sequencing reads from resequenced products were merged for all subsequent analyses to maintain consistency between bacterial and fungal analyses. Additionally, combining data from resequenced samples likely increased sampling depth – for PCR replication, prior work has shown that combining data from replicates increases the ability to accurately sample the diversity and relative read abundances of taxa within a given microorganism community.^113^

##### Phyloseq objects for each research aim

For further analysis, the bacterial and fungal phyloseq objects were subset by research aim, samples with <1,000 sequencing reads were discarded, and phyloseq objects were rarefied to the minimum read depth (see rarefaction curves in Figures S5–S10). The final bacterial and fungal objects for Aim 1 consisted of data from feather samples from all three sparrow hosts collected during the June spring tide. The final bacterial and fungal objects for Aim 2 consisted of data from Maine Nelson’s sparrow feather samples. Due to variation in bacterial and fungal sediment sample sequence read quality, the final bacterial and fungal objects for Aim 3 differed in their data composition. The final bacterial object for Aim 3 consisted of data from Maine Nelson’s sparrow feather and Maine sediment samples; the sediment samples collected during the June spring tide were not included due to low sample size after removing samples with <1,000 reads. In contrast, the final fungal object for Aim 3 included data from all feather and all sediment samples. All objects were made again with a subset of bacterial and fungal genera known to contain species with keratinolytic properties, as identified through a literature search (Tables S15 and S16).

#### Statistical analyses

##### Alpha diversity and community composition

For all research aims, variation in alpha diversity and community composition was investigated across our variables of interest, as follows: Aim 1: sparrow host species; Aim 2: sex, marsh, month in the breeding season, tidal cycle, and tidal range within eastern Maine Nelson’s feather samples; Aim 3: feather and sediment sample types. Figure 2 outlines the research questions and corresponding statistical methods for each research aim.

For both bacterial and fungal data, our metrics of alpha diversity, observed richness (number of SVs) and Shannon’s diversity, were estimated using the “estimate_richness()” function within *phyloseq*. Significance of variation in diversity metrics among groups was tested using analysis of variance or Kruskal-Wallis tests (for data with non-normal residuals). *Post-hoc* Tukey’s Honest Significant Differences (TukeyHSD) tests were used to identify pairwise differences in alpha diversity. Similarity in community composition among samples was visually assessed using Principal Coordinate Analysis (PCoA) performed with Jaccard dissimilarity (considers presence/absence of taxa) as well as Bray-Curtis dissimilarity (considers relative abundance of taxa). Permutational multivariate analysis of variance (PERMANOVA) within the vegan package^112^ and *post-hoc* pairwise PERMANOVA within the pairwiseAdonis package^114^ were performed with Jaccard and Bray-Curtis dissimilarity to statistically test for variation in community composition among groups. PERMANOVA results indicate if there are differences in centroids among groups (indicating significantly different between-group community composition), but are sensitive to variance in dispersion between groups (the average distance of group members to the centroid, i.e., degree of variability in community composition within a group.^115^ To identify comparisons where variance in group dispersion may influence PERMANOVA results, homogeneity of variance tests (“betadisperser()” and “permutest()” functions within the *vegan* package) and *post-hoc* TukeyHSD tests were performed. In cases where unbalanced sample sizes led to deviations from homogeneity of variance, PERMANOVA results are not reported. To further compare community composition, DESeq^27^ was used to identify differentially abundant taxa between groups. Taxa were considered differentially abundant at a Benjamin-Hochberg corrected p-value of 0.01.

##### Composition of taxa between feather and sediment samples

For Aim 3, the assessment of variation between plumage and sediment samples, we performed two additional analyses. We identified taxa shared across both sample types as well as the “core” (present in a large percentage of samples) and “peripheral” (present only in a small percentage of samples) taxa for each sample type (sediment or plumage). These additional analyses were performed after initial investigations revealed marked differences in alpha diversity and community composition between plumage and sediment bacteria communities. Specifically, we were interested in identifying those taxa that were characteristic of each sample type (i.e., taxa that were common in one sample type *and* not shared between both sample types).

The core and peripheral microbiota of unrarefied datasets were identified using the “core_members()” and “rare()” functions within the package *microbiome*.^116^ Two sets of core and peripheral communities were defined, the first using a ≥50% prevalence for inclusion in the core community and a ≤10% prevalence for inclusion in the peripheral community. The second set used a ≥60% core prevalence and a ≤20% peripheral prevalence. All communities were defined using a detection threshold of 0.001%. While no consensus exists across the literature on what percentage of samples in which taxa must occur to be considered part of the “core” community, ranges between 50-100% occurrence are commonly used.^117^ The package *ggVennDiagram*^118^ was then used to identify the number of taxa shared between unrarefied plumage and sediment samples, using no cutoffs for prevalence or detection thresholds. These methods were used to analyze both bacteria and fungi sequence data, but with only eastern Maine plumage and eastern Maine sediment samples for bacteria (due to sample size restrictions) and all plumage and sediment samples for fungi.

## Supplemental information

Document S1. Figures S1–S10 and Tables S1–S10 and S12–S16

Table S11. Differentially abundant Nelson’s sparrow plumage bacterial and fungal sequence variants across variables, related to Figure 8 Differentially abundant SVs were detected by DESeq at a significance level of p < 0.01.
