# Supplementary material for: Plumage microorganism communities of tidal marsh sparrows
Source: iScience. 2023 Dec 7;27(1):108668. doi: 10.1016/j.isci.2023.108668 (PMC10790016; doi:10.1016/j.isci.2023.108668)
Supplement: Document S1. Figures S1–S10 and Tables S1–S10 and S12–S16 [file mmc1.pdf]

iScience, Volume *27*

Supplemental information

Plumage microorganism communities

# of tidal marsh sparrows

Alice M. Hotopp, Brian J. Olsen, Suzanne L. Ishaq, Serita D. Frey, Adrienne I. Kovach, Michael T. Kinnison, Franco N. Gigliotti, Mackenzie R. Roeder, and Kristina M. Cammen

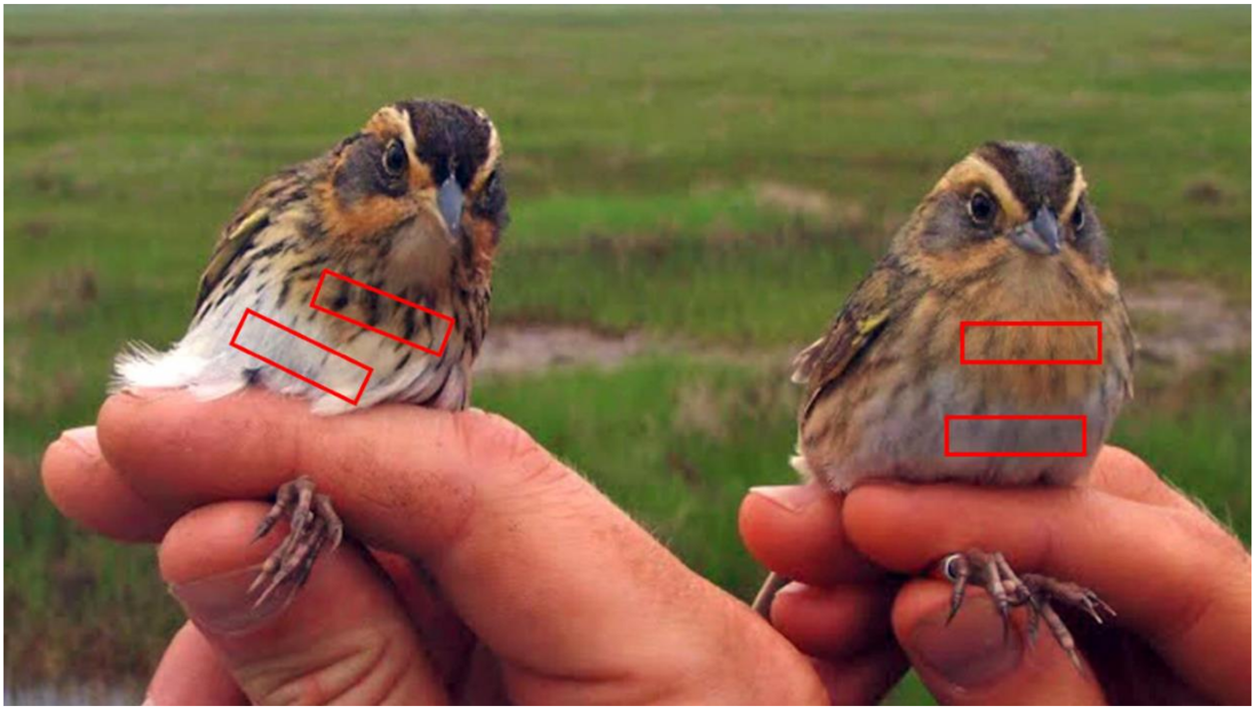

Figure S1. Photo of a saltmarsh sparrow (left) and a Nelson's sparrow (right), related to STAR Methods. Red boxes outline the two regions of the breast (the upper, streaky region and the lower, white region) from where feathers were sampled in order to investigate the impact of feather color on microorganism communities.

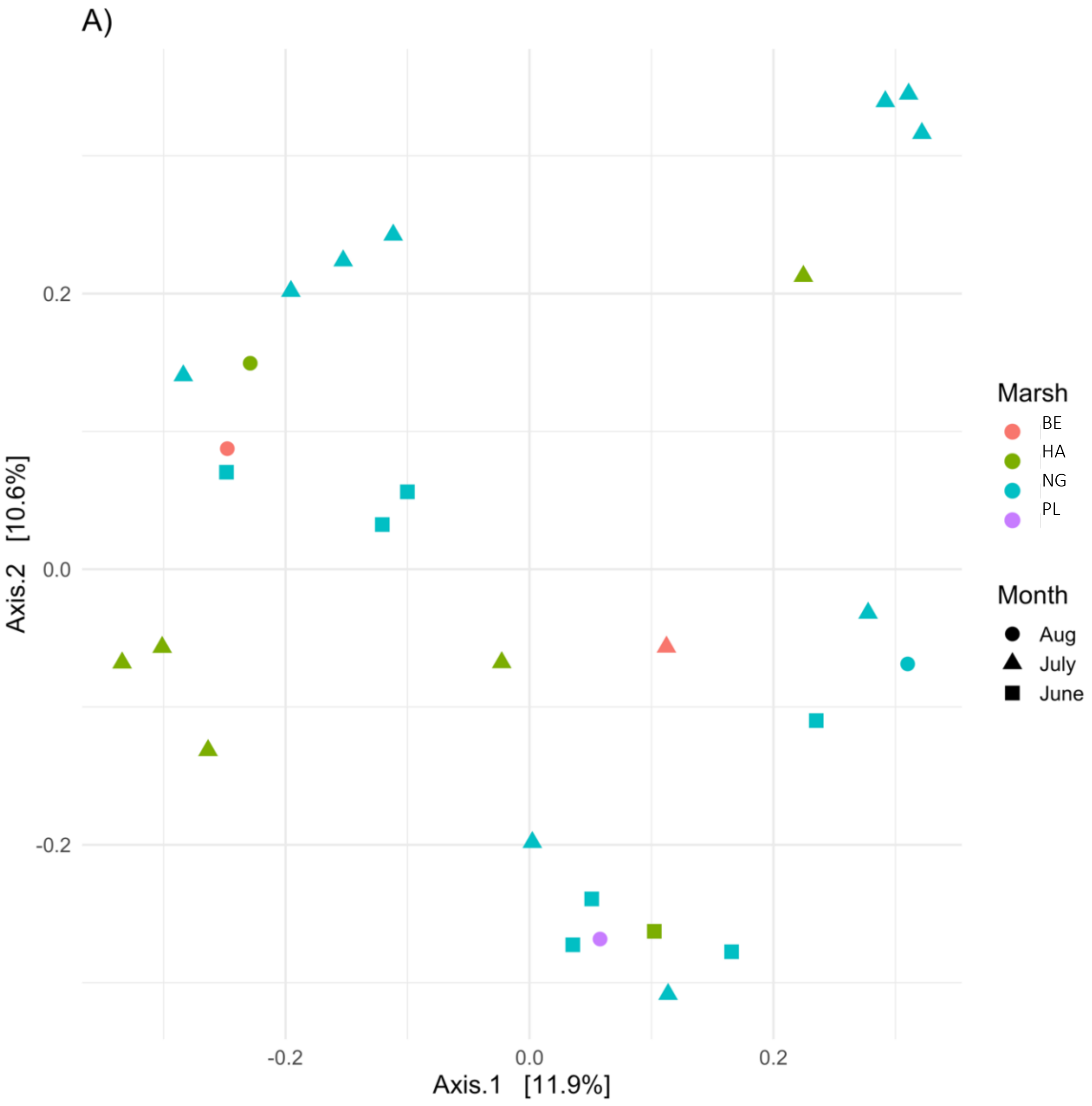

Figure S2. Within-species plumage bacteria community composition, related to Figure 7. PCoA using Jaccard dissimilarity of plumage bacterial communities of eastern Maine Nelson's sparrows (n = 28) across marsh and month.

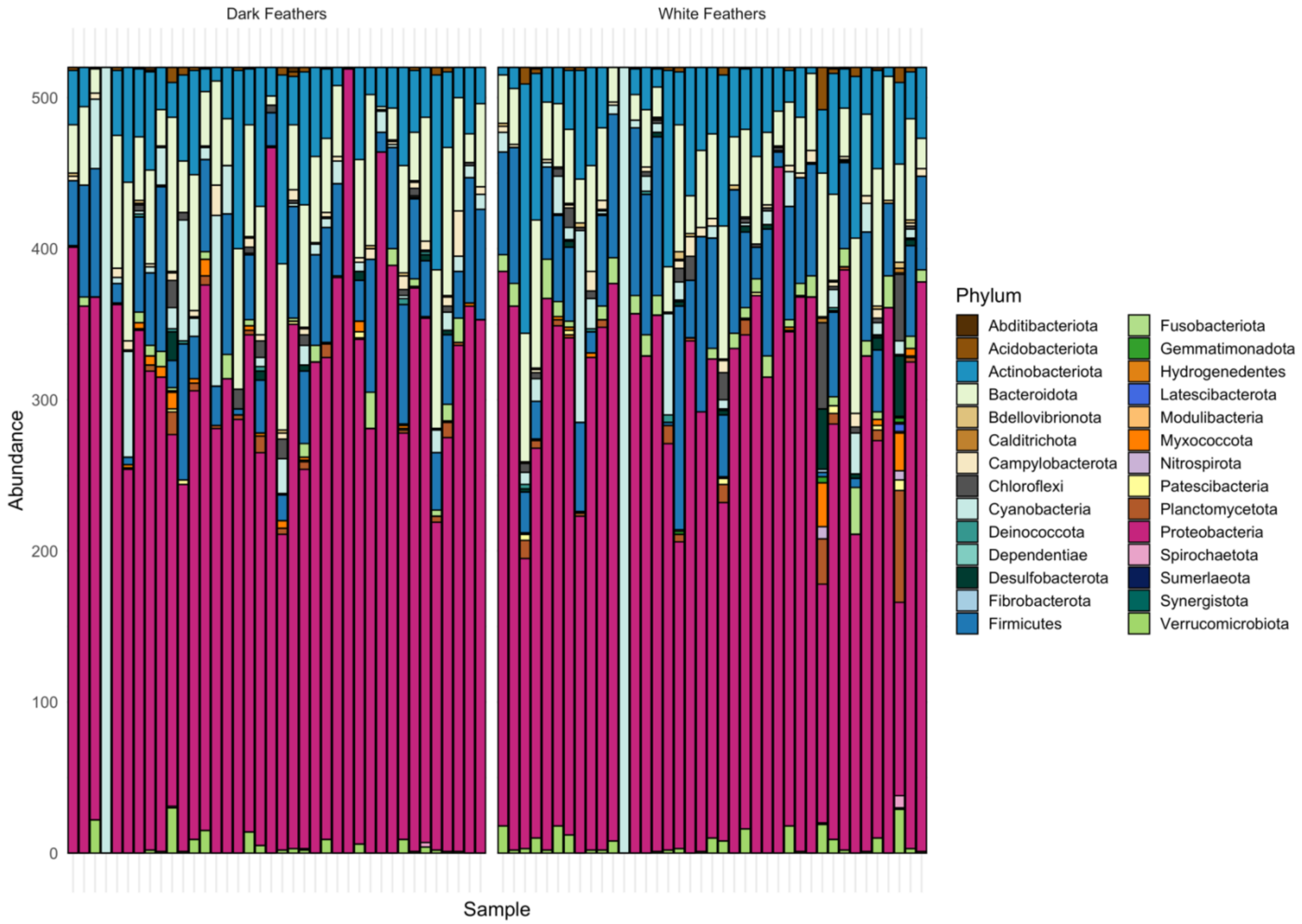

Figure S3. Comparison of relative abundance of bacterial phyla between feather colors, related to STAR Methods. Bacterial phyla inhabiting dark feathers on the left, those inhabiting white feathers on the right.

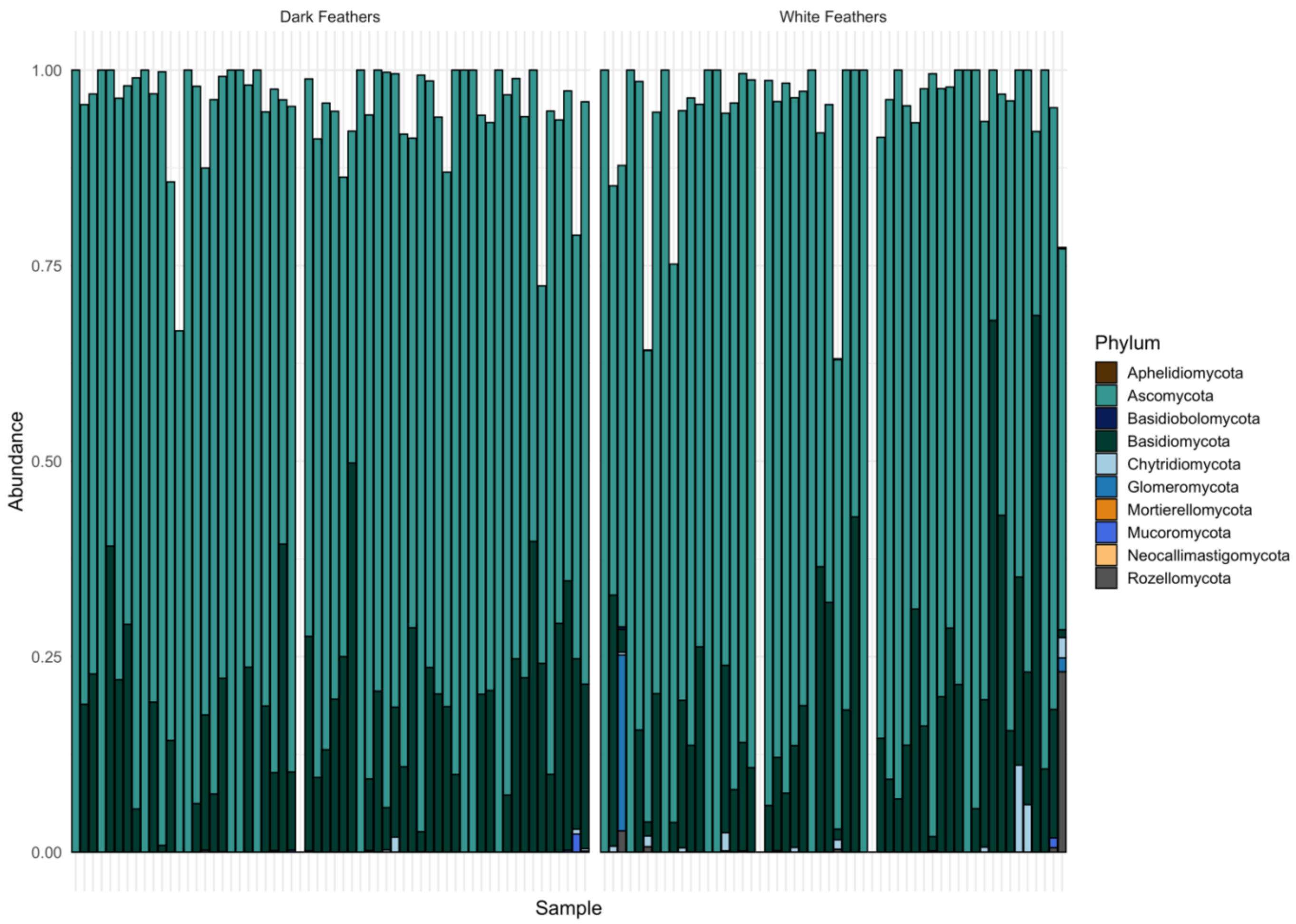

Figure S4. Comparison of relative abundance of fungal phyla between feather colors, related to STAR Methods. Fungal phyla inhabiting dark feathers on the left, those inhabiting white feathers on the right.

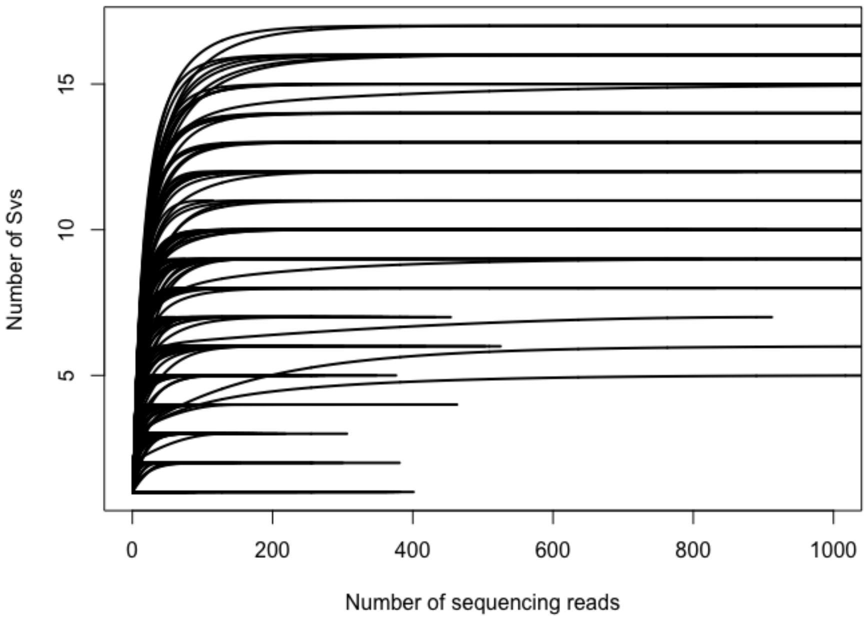

Figure S5. Rarefaction curve, 16S rRNA sequences from tidal marsh sparrow plumage samples, related to STAR Methods.

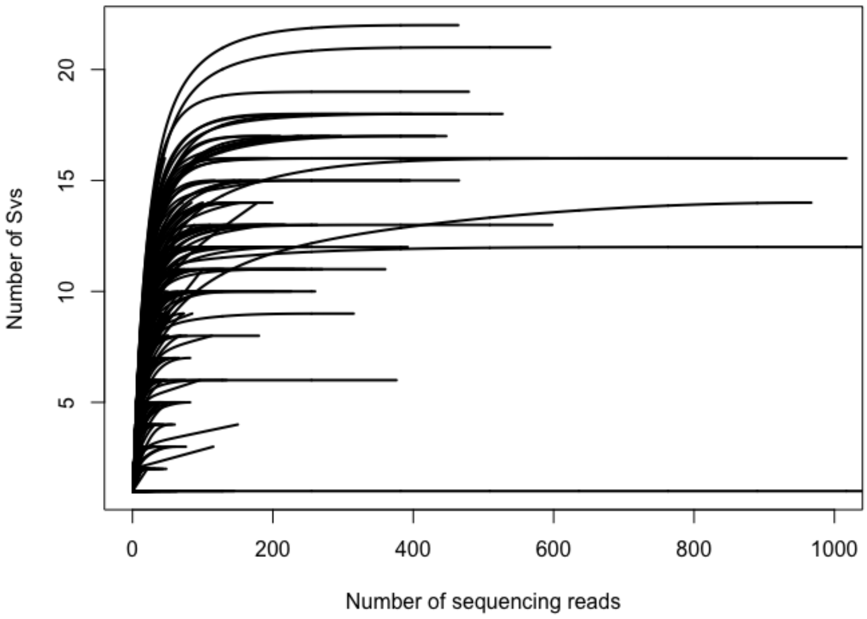

Figure S6. Rarefaction curve, 16S rRNA sequences from Maine Nelson's sparrow plumage samples, related to STAR Methods.

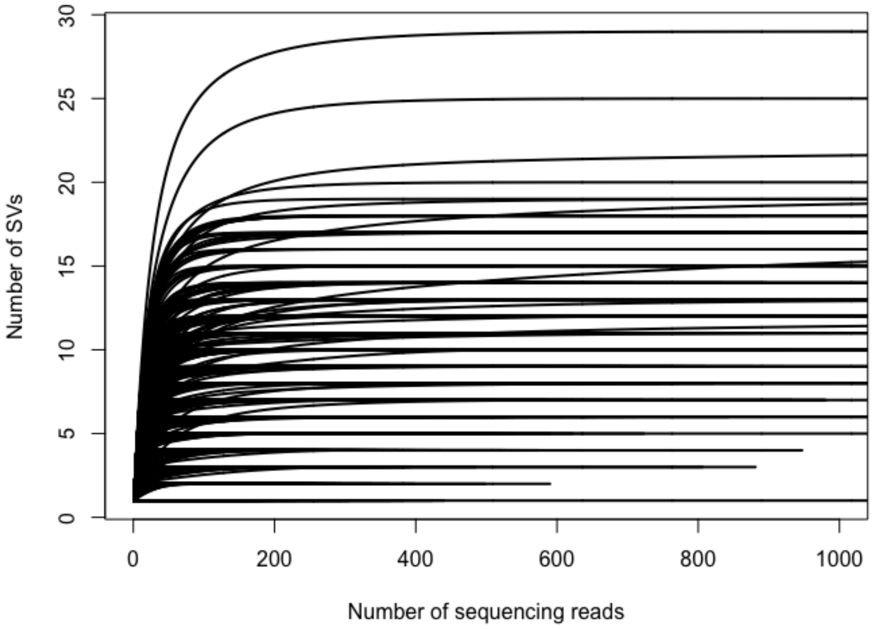

Figure S7. Rarefaction curve, 16S rRNA sequences from Maine Nelson's sparrow plumage and Maine sediment samples, related to STAR Methods.

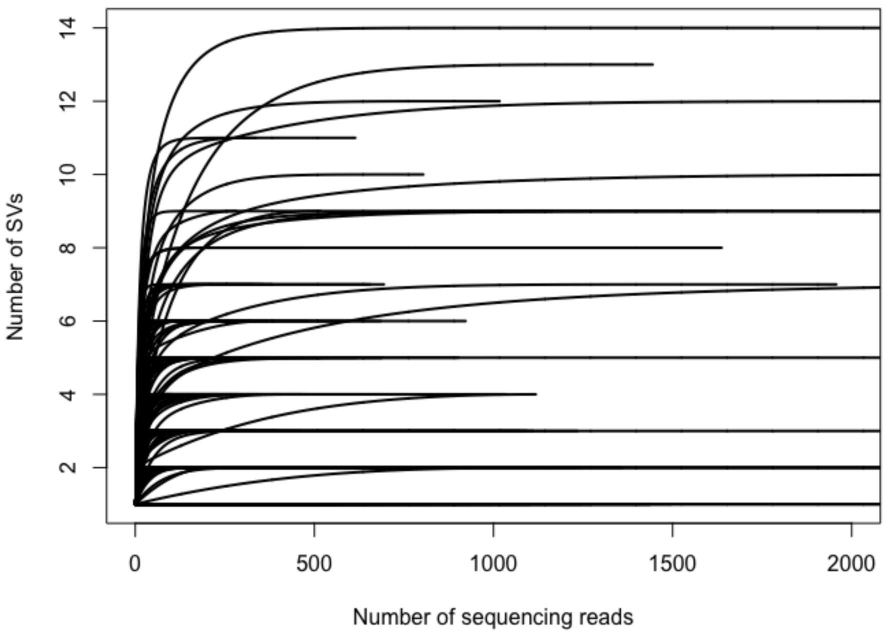

Figure S8. Rarefaction curve, ITS sequences from tidal marsh sparrow plumage samples, related to STAR Methods.

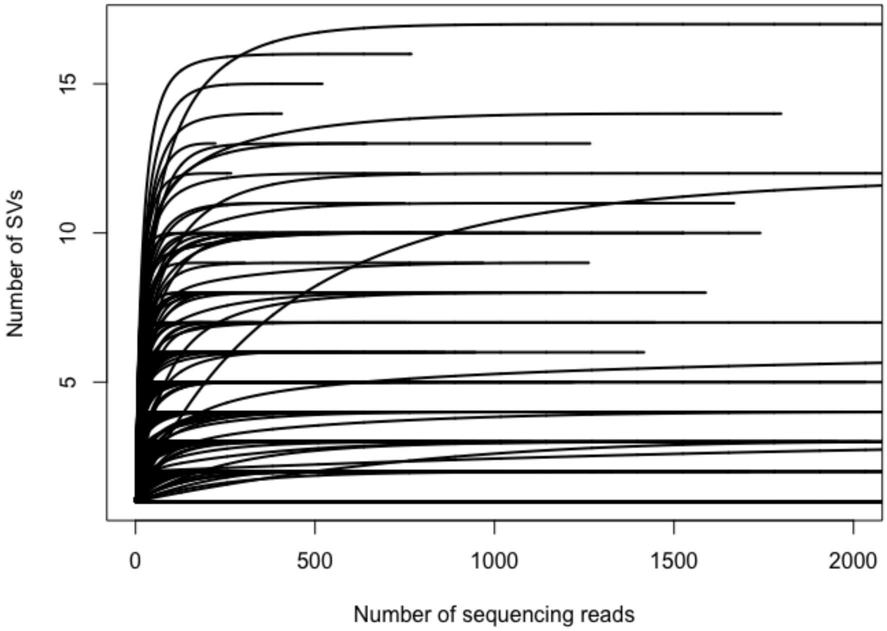

Figure S9. Rarefaction curve, ITS sequences from Maine Nelson's sparrow plumage samples, related to STAR Methods.

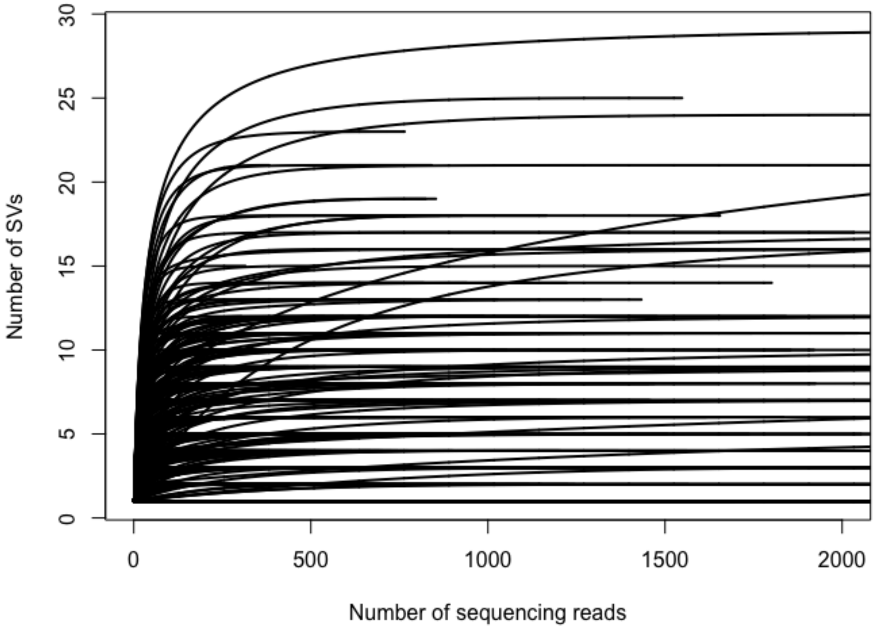

Figure S10**.** Rarefaction curve, ITS sequences from tidal marsh sparrow plumage and sediment samples, related to STAR Methods.

Table S1. Effect of variables on within-host species observed bacterial diversity, related to Figures 3 & 6. Kruskal-Wallis results of the effect of sex, marsh, month, tidal cycle, and tidal range on bacterial taxa observed diversity across Downeast ME Nelson's sparrow plumage samples.

Variable	DF	X^2	P-value
Sex	1, 26	0.52978	0.4667
Marsh	3, 24	3.9395	0.2681
Month	2, 25	2.4883	0.2882
Tidal cycle	1, 26	1.0229	0.3118
Tidal range	4, 23	5.9255	0.2048

Table S2. Effect of variables on within-host species Shannon bacterial diversity, related to Figures 3 & 6. Kruskal-Wallis results of the effect of sex, month, tidal cycle, and tidal range on bacterial taxa Shannon diversity across Downeast ME Nelson's sparrow plumage samples.

Variable	DF	X^2	P-value
Sex	1, 26	0.2006	0.6542
Marsh	3, 24	3.8259	0.2809
Month	2, 25	1.621	0.4446
Tidal cycle	1, 26	0.84142	0.359
Tidal range	4, 23	4.2038	0.3791

Table S3. Effect of variables on within-host species observed diversity of keratinolytic bacterial genera, related to Figure 6. Kruskal-Wallis results of the effect of sex and tidal cycle on observed diversity of the subset of bacterial genera containing keratinolytic taxa across Downeast ME Nelson's sparrow plumage samples.

Variable	DF	X^2	P-value
Sex	1, 25	2.6872	0.1012
Tidal cycle	1, 25	1.3551	0.24444

Table S4. Effect of variables on within-host species observed diversity of keratinolytic bacterial genera, related to Figure 6. ANOVA results of the effect of marsh and month on observed diversity of the subset of bacterial genera containing keratinolytic taxa across Downeast ME Nelson's sparrow plumage samples.

Variable	DF	F-value	P-value
Marsh	3, 23	0.395	0.758
Month	2, 24	1.637	0.215
Tidal range	4, 22	1.42	0.26

Table S5. Effect of variables on within-host species Shannon diversity of keratinolytic bacterial genera, related to Figure 6. ANOVA results of the effect of sex, marsh, month, tidal cycle, and tidal range on Shannon diversity of the subset of bacterial genera containing keratinolytic taxa across Downeast ME Nelson's sparrow plumage samples.

Variable	DF	F-value	P-value
Sex	1, 25	3.189	0.0863
Marsh	3, 23	0.385	0.765
Month	2, 24	2.806	0.0804
Tidal cycle	1, 25	2.95	0.0983
Tidal range	4, 22	0.2374	0.231

Table S6. Effect of variables on within-host species observed fungal diversity, related to Figures 3 & 6. ANOVA results of the effect of sex, marsh, month, and tidal cycle on fungal taxa observed diversity across Downeast ME Nelson's sparrow plumage samples.

Variable	DF	F-value	P-value
Sex	1, 35	2.453	0.126
Marsh	3, 33	1.553	0.219
Month	2, 34	0.97	0.39
Tidal cycle	1, 35	1.672	0.204
Tidal range	4, 32	0.813	0.526

Table S7. Effect of variables on within-host species Shannon fungal diversity, related to Figures 3 & 6. ANOVA results of the effect of sex, marsh, month, and tidal cycle on fungal taxa Shannon diversity across Downeast ME Nelson's sparrow plumage samples.

Variable	DF	F-value	P-value
Sex	1, 35	1.075	0.307
Marsh	3, 33	2.244	0.102
Month	2, 34	0.96368	0.2637
Tidal cycle	1, 35	1.903	0.177
Tidal range	4, 32	1.239	0.314

Table S8. Effect of variables on within-host species observed diversity of keratinolytic fungal genera, related to Figure 6. Kruskal-Wallis results of the effect of sex, marsh, month, and tidal cycle on fungal taxa observed diversity of the subset of fungal genera containing keratinolytic taxa across Downeast ME Nelson's sparrow plumage samples.

Variable	DF	X^2	P-value
Sex	1, 31	0.0041121	0.9489
Marsh	3, 29	0.27737	0.9642
Month	2, 30	4.2988	0.1166
Tidal cycle	2, 30	12.5228	0.2833
Tidal range	3, 29	2.4952	0.4762

Table S9. Effect of variables on within-host species Shannon diversity of keratinolytic fungal genera, related to Figure 6. ANOVA results of the effect of sex, marsh, month, and tidal cycle on fungal taxa Shannon diversity of the subset of fungal genera containing keratinolytic taxa across Downeast ME Nelson's sparrow plumage samples.

Variable	DF	F-value	P-value
Sex	1, 31	0.316	0.578
Marsh	3, 29	0.303	0.823
Month	2, 30	1.007	0.354
Tidal cycle	2, 30	1.142	0.333
Tidal range	3, 29	0.234	0.872

Table S10. Within-host species bacterial community composition, related to Figures 5 & 7. PERMANOVA results for assessing variation in bacterial community composition across sex, month, and tidal range in Downeast ME Nelson's sparrow plumage samples.

Variable	DF	R^2	F-Value	P-Value
Sex				
Jaccard	1, 26	0.03985	1.0791	0.272
Bray-Curtis	1, 26	0.04207	1.1419	0.261
Month				
Jaccard	2, 25	0.08492	1.16	0.132
Bray-Curtis	2, 25	0.08602	1.1765	0.168
Tidal Range				
Jaccard	2, 25	0.16696	1.1524	0.08
Bray-Curtis	2, 25	0.17214	1.1956	0.119

Table S12. Within-host species fungal community composition, related to Figures 5 & 7. PERMANOVA results for assessing variation in community composition across the subset of fungal genera containing keratinolytic taxa in Downeast ME Nelson's sparrow plumage samples.

Variable	DF	R^2	F-Value	P-Value
Marsh				
Jaccard	3, 29	0.09171	0.976	0.532
Bray-Curtis	3, 29	0.09137	0.972	0.549
Month				
Jaccard	2, 30	0.5682	0.9037	0.692
Bray-Curtis	2, 30	0.05428	0.8609	0.717
Tidal cycle				
Jaccard	2, 30	0.07278	1.1774	0.133
Bray-Curtis	2, 30	0.07724	1.2555	0.12
Tidal range				
Jaccard	3, 29	0.08561	0.9051	0.743
Bray-Curtis	3, 29	0.08343	0.8799	0.734

Table S13. Differentially abundant fungal taxa in feather and sediment samples, related to Figure 8. Differentially abundant SVs were detected by DESeq at a significance level of $p < 0.01$.

Sample type comparison	SVs more abundant in feathers than sediment	Taxa	Absolute value log2fold change	BH corrected p-value
Feather versus sediment	14			
		Symmetrospora symmetrica	23.41	3.1E-11
		Neodevriesiaceae SV	23.23	3.1E-11
		Sordariomycetes	23.15	3.1E-11
		Neodevriesiaceae SV	23.13	3.1E-11
		Erythrobasidium hasegawianum	22.99	3.2E-11
		Papiliotrema taeanensis	22.81	3.2E-11
		Genolevuria SV	22.78	3.2E-11
		Papiliotrema mangalensis	22.72	3.2E-11
		Sarocladium SV	22.70	3.2E-11
		Symmetrospora symmetrica	22.68	3.2E-11
		Magnaporthaceae	22.18	8.6E-11
		Neodevriesiaceae	21.85	1.5E-10
		Dioszegia SV	21.84	1.5E-10
		Lachnum spartinae	9.04	1.9E-03

Table S14. Summary of reads lost during pre-processing steps, related to STAR Methods. Total reads, reads removed during filtering and chimera-removal steps, and remaining reads from each of four 1S6 rRNA and ITS sequencing runs.

	Total reads	No. removed during filtering	No. chimeric reads removed	Remaining reads
**16S**				
Feathers n = 259				
Sediment n = 32				
Extract control n = 22				
PCR control n = 25				
Run 1	8,092,437	570,497	319,484	7,202,456
Run 2	5,017,248	211,409	302,863	4,502,976
Run 3	7,135,112	518,255	148,297	6,468,560
Run 4	4,260,316	210,413	285,515	3,764,388
**ITS**				
Feathers n = 279				
Sediment n = 27				
Extract control n = 21				
PCR control n = 25				
Run 1	1,789,648	288,350	74,648	1,426,650
Run 2	3,435,594	741,229	1,255,516	1,438,849
Run 3	5,835,524	887,633	329,902	4,617,989
Run 4	2,079,824	236,443	108,750	1,734,631

Table S15. Bacterial genera containing keratinolytic taxa, related to table 4 and STAR Methods. Taxa were identified through a literature search.

Genus
Bacillus[1]
Streptomyces[1]
Pseudomonas[2]
Enterococcus[2]
Staphylococcus[2]
Kocuria[2]
Arthrobacter[3]
Fervidobacterium[3]
Janthinobacterium[3]
Alcaligenes[3]
Terrabacter[3]
Nesterenkonia[3]
Caldicoprobacter[3]
Thermoanaeobacter[3]
Flevibacterium[3]
Actinomadura[3]
Amylocolatopsis[3]
Brevibacillus[3]
Fervidobacterium[3]
Nocardiosis[3]
Saccharomonospara[3]
Serratia[3]
Thermoactinomyces[3]
Flavobacterium[4]
Chryseobacterium[5]
Stenotrophomonas[6]
Xanthomonas[6]
Microbacterium[6]
Paenibacillus[6]

Table S16. Fungal genera containing keratinolytic taxa, related to Table 4 and STAR Methods. Taxa were identified through a literature search.

Genus
Aspergillus[7]
Trychophyton[7]
Doratomyces[7]
Onygena[7]
Arthroderma[7]
Microsporum[7]
Coccidoides[7]
Gymnoascoideus [7]
Talaromyces[7]
Paecilomyces[7]
Scopulariopsi[7]
Myrothecium[7]
Tritirachium[7]
Trichoderma[7]
Candida[7]
Geotrichum[7]
Engyodontium[7]
Myrothecium[7]
Cadophora[8]
Neosetophoma[8]
Pseudogymnoascus[8]
Oidiodendron[9]
Chrysosporium[9]
Penicillium[9]
Curvularia[10]
Keratinomyces[10]
Acrodontium[10]
Botryotricum[10]
Chaetomium[10]
Chrysosporium[10]
Gliocladium[10]
Keratinophyton[10]
Malbranhea[10]
Microsporum[10]
Verticillium[10]
Auxarthron[10]
Alternaria[10]
Trichurus[10]
Geomyces[10]
Gleomastis[10]
Monodictys[10]
Stachybotrys[10]
Urocladium[10]

Aphanoascus[10]
Fusarium[11]
Cladosporium[11]
Phytophthora[11]
Sepedonium[12]
Aphanoascus[12]
Myriodontium[12]
Gymnoascus[13]

Literature Cited

1. Sivakumar, N., and Raveendran, S. (2015). Keratin degradation by bacteria and fungi isolated from a poultry farm and plumage. http://dx.doi.org.wv-o-ursus-proxy02.ursus.maine.edu/10.1080/00071668.2014.996119 *56*, 210–217. 10.1080/00071668.2014.996119.
2. Kowalczyk, P., Mahdi-Oraibi, S., Misiewicz, A., Gabzdyl, N., Miskiewicz, A., and Szparecki, G. (2018). Feather-Degrading Bacteria: Their Biochemical and Genetic Characteristics. Arab. J. Sci. Eng. *43*, 33–41. 10.1007/s13369-017-2700-2.
3. Sharma, R., and Devi, S. (2018). Versatility and commercial status of microbial keratinases: a review. Rev. Environ. Sci. Bio/Technology *17*, 19–45. 10.1007/s11157-017-9454-x.
4. Riffel, A., and Brandelli, A. (2002). Isolation and characterization of a feather-degrading bacterium from the poultry processing industry. J. Ind. Microbiol. Biotechnol. *29*, 255–258. 10.1038/sj.jim.7000307.
5. Riffel, A., Lucas, F., Heeb, P., and Brandelli, A. (2003). Characterization of a new keratinolytic bacterium that completely degrades native feather keratin. Arch. Microbiol. *179*, 258–265. 10.1007/S00203-003-0525-8/TABLES/3.
6. Nasipuri, P., Herschend, J., Brejnrod, A.D., Madsen, J.S., Espersen, R., Svensson, B., Burmølle, M., Jacquiod, S., and Sørensen, S.J. (2020). Community-intrinsic properties enhance keratin degradation from bacterial consortia. PLoS One *15*, e0228108. 10.1371/JOURNAL.PONE.0228108.
7. Lange, L., Huang, Y., and Busk, P.K. (2016). Microbial decomposition of keratin in nature-a new hypothesis of industrial relevance. Appl. Microbiol. Biotechnol. *100*, 2083–2096. 10.1007/s00253-015-7262-1.
8. Robicheau, B.M., Adams, S.J., Provencher, J.F., Robertson, G.J., Mallory, M.L., and Walker, A.K. (2019). Diversity and keratin degrading ability of fungi isolated from Canadian arctic marine bird feathers. Arctic *72*, 347–359. 10.14430/arctic69301.
9. Pugh, G.J.F. (1965). Cellulolytic and keratinophilic fungi recorded on birds. Sabouraudia *4*, 85–91. 10.1080/00362176685190221.
10. Singh, I., and Kushwaha, R.K.S. (2015). Keratinases and microbial degradation of Keratin. Adv. Appl. Sci. Res. *6*, 74–82.
11. Călin, M., Constantinescu-Aruxandei, D., Alexandrescu, E., Răut, I., Doni, M.B., Arsene, M.L., Oancea, F., Jecu, L., and Lazăr, V. (2017). Degradation of keratin substrates by keratinolytic fungi. Electron. J. Biotechnol. *28*, 101–112. 10.1016/J.EJBT.2017.05.007.
12. Nardoni, S., and Mancianti, F. (2021). Survey of Keratinophilic Fungi from Feathers of

Birds in Tuscany. Biology (Basel). *10*, 1317. 10.3390/biology10121317.
13. Deshmukh, S.K., and Verekar, S.A. (2010). Incidence of keratinophilic fungi from the soils of Vedanthangal Water Bird Sanctuary (India). Mycoses *54*, 487–490. 10.1111/j.1439-0507.2010.01882.x.
